# Deletion of MMP12 improves energy metabolism and brown adipose tissue function in mice prone to cardiometabolic disease

**DOI:** 10.1016/j.jlr.2025.100951

**Published:** 2025-11-26

**Authors:** Melina Amor, Malena Diaz, Alexander Fuerlinger, Monika Svecla, Valentina Bianco, Laszlo Schooltink, Anja Dobrijević, Birgit Schwarz, Alena Akhmetshina, Nemanja Vujić, Melanie Korbelius, Martin Hirtl, Martin Buerger, Anita Pirchheim, Silvia Rainer, Silvia Schauer, Giangiacomo Beretta, Walter Goessler, Dagmar Kolb, Gerald Hoefler, Hubert Hackl, Corina Madreiter-Sokolowski, Mahmoud Abdellatif, Giuseppe Danilo Norata, Dagmar Kratky

**Affiliations:** 1 Molecular Biology and Biochemistry, Gottfried Schatz Research Center, Medical University of Graz, Graz, Austria; 2 Department of Cardiology, Medical University of Graz, Graz, Austria; 3 Department of Pharmacological and Biomolecular Sciences, University of Milan, Milan, Italy; 4 Diagnostics and Research Institute of Pathology, Medical University of Graz, Graz, Austria; 5 Department of Environmental Science and Policy, Università degli Studi di Milano, Milan, Italy; 6 Institute of Analytical Chemistry, University of Graz, Graz, Austria; 7 Cell Biology, Histology and Embryology, Gottfried Schatz Research Center, Medical University of Graz, Graz, Austria; 8 Core Facility Ultrastructural Analysis, Medical University of Graz, Graz, Austria; 9 BioTechMed-Graz, Graz, Austria; 10 Institute of Bioinformatics, Medical University of Innsbruck, Innsbruck, Austria

**Keywords:** Adipose tissue, brown, Extracellular matrix, Inflammation, Mitochondria, Proteomics

## Abstract

Matrix metalloproteinase-12 (MMP12) is a proinflammatory macrophage-secreted protein with immunomodulatory functions that affects neutrophil infiltration, cytokine release, macrophage recruitment, and proliferation. We have previously demonstrated that the genetic deletion of MMP12 in a cardiometabolic mouse model ameliorates obesity-induced low-grade inflammation, white adipose tissue dysfunction, and atherosclerosis. Based on the various beneficial metabolic effects of MMP-12 deletion, we hypothesized that loss of MMP-12 also positively affects whole-body energy metabolism and/or brown adipose tissue (BAT) function in a cardiometabolic mouse model. To investigate the effects of MMP12 deletion on whole-body energy metabolism and/or BAT function, we used low-density lipoprotein receptor (Ldlr)/Mmp12 double knockout (DKO) fed a high-fat, sucrose- and cholesterol-enriched diet. DKO mice housed at 22°C showed increased energy expenditure and decreased BAT size and triglyceride (TG) content. Untargeted proteomic analyses revealed the upregulation of proteins and pathways related to mitochondrial function, glucose metabolism, and fatty acid oxidation in the BAT of DKO mice, whereas the abundance of proteins and pathways associated with inflammation was reduced. In addition, DKO mice exhibited reduced macrophage infiltration in BAT, with the infiltrating macrophages showing lower expression of lipid-associated marker genes. Loss of MMP12 was associated with reduced compactness and sphericity of the mitochondria in the BAT. Following an acute cold exposure, DKO mice had decreased circulating lipid concentrations, especially very low-density lipoprotein-TG and LDL-cholesterol, and increased expression of thermogenic genes. We conclude that MMP12 may play a detrimental role in whole-body energy homeostasis and thermogenesis, as it triggers macrophage infiltration, inflammation, and mitochondrial dysfunction in BAT.

## Highlights

• MMP12 deletion is linked to inflammation-related pathways in brown adipose tissue.
• Reduced macrophages in the brown adipose tissue of Mmp12-deficient mice.
• Improved energy expenditure in cardiometabolic Mmp12-deficient mice.
• MMP12 is expressed in the mitochondrial fraction of brown adipose tissue.
• Cold exposure exacerbates the effects of MMP12 deficiency in brown adipose tissue.

## Graphical Abstract

**Figure:**
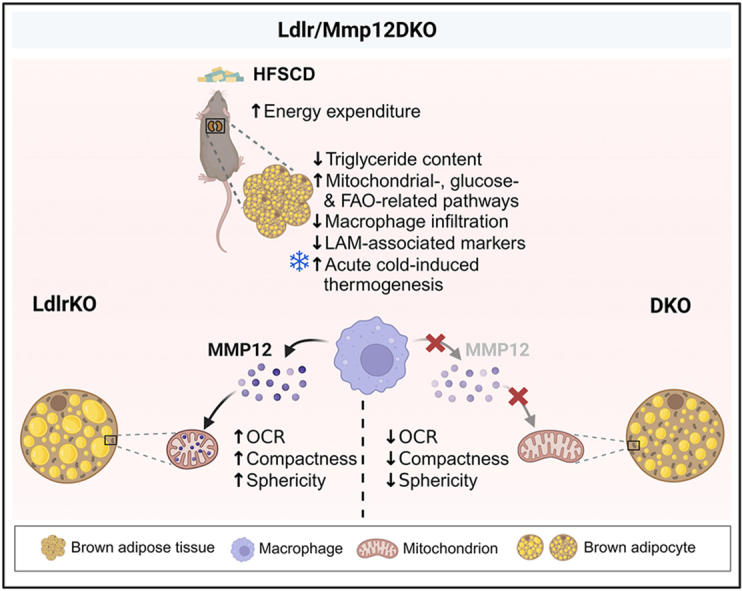

Brown adipose tissue (BAT) is a thermogenic organ responsible for non-shivering heat production, primarily mediated by uncoupling protein 1 (UCP1) in the mitochondrial inner membrane. UCP1 dissipates the proton gradient across the inner mitochondrial membrane, uncoupling oxidative phosphorylation from ATP production to generate heat (1). Cold exposure is the most extensively studied physiological stimulus to induce BAT activation, leading to the release of norepinephrine by the sympathetic nervous system, which in turn activates β-adrenergic signaling and UCP1 (2). In addition to thermogenesis, BAT activation is also associated with elevated triglyceride (TG)-rich lipoprotein turnover, glucose uptake, the breakdown of branched-chain amino acids, which leads to antioxidant protection, and the secretion of batokines with beneficial autocrine, paracrine, and endocrine effects (3, 4, 5, 6). The presence and activation of BAT in humans is associated with improved cardiometabolic health, including lower prevalence of type 2 diabetes, dyslipidemia, and cardiovascular diseases (7). Thus, increasing BAT mass and activity could be a potential therapeutic strategy to improve metabolic health and combat cardiometabolic diseases.

Matrix metalloproteinases (MMPs) are a family of zinc-dependent endopeptidases that play crucial roles in extracellular matrix (ECM) remodeling and degradation, including the cleavage of proteins of the complement and blood clotting system, cell adhesion molecules, cytokines, chemokines, and angiogenesis-related proteins (8). Due to this broad substrate specificity, MMPs are well known to both liberate ECM-derived growth factors and cytokines and to alter the signaling, migration, differentiation, proliferation, and apoptosis of multiple cell types (9). Thus, dysregulated MMP activity leads to numerous pathological conditions such as arthritis, inflammation, cancer, and atherosclerosis, highlighting MMPs as promising therapeutic targets. The fact that certain members of the MMP family have diverse proteolytic and non-proteolytic activities in various intracellular compartments opened up a new field of research on MMPs (10).

Elevated levels of MMP12, a multifaceted protein secreted by macrophages, have been proposed as an unfavorable prognostic marker in various inflammatory and metabolic pathologies, making MMP12 an attractive molecular target (11, 12, 13). MMP12 exhibits immunomodulatory effects by influencing neutrophil infiltration, cytokine release, macrophage recruitment, and proliferation (14, 15, 16). After binding to cellular membranes, MMP12 is internalized within minutes or even seconds, depending on the cell type (17). Independent of its proteolytic functions, MMP12 was shown to act as a transcription factor that stimulates antiviral immunity (18). The carboxyterminal domain of MMP12 promotes bacterial cell death of phagocytosed bacteria (19). MMP12 is also regarded as a marker for a particular subtype of tissue-resident macrophages, designated as lipid-associated macrophages (LAMs), which have been identified in multiple tissues under various metabolic conditions (20). In diet-induced obese mice, LAM abundance is increased in BAT, where these cells reduce the thermogenic capacity of BAT and drive its transition to a white adipose tissue (WAT) phenotype (21). MMP12 was described as a negative regulator of glucose metabolism, causing mitochondrial dysfunction and insulin resistance in vitro in adipocytes from Western-type diet-fed mice. (22). In line with this, MMP12 siRNA improves high-fat diet-induced metabolic disorders and intestinal homeostasis (23). In addition, genetic deletion of MMP12 ameliorates cardiometabolic diseases by reducing circulating lipid levels, obesity-induced low-grade inflammation, WAT dysfunction, insulin sensitivity, and atherosclerosis in a room temperature-housed cardiometabolic mouse model (24). Along with the beneficial changes in the WAT proteome of these mice, we therefore hypothesized that MMP12 also plays a detrimental role in BAT by affecting its function and inflammatory processes.

## Materials and Methods

### Animals and diet

Low-density lipoprotein receptor (Ldlr)/Mmp12-double knockout (DKO) mice were generated by breeding Mmp12KO with LdlrKO mice (both from The Jackson Laboratory). Male and female Ldlr^+/−^Mmp12^+/−^ mice were crossed to produce LdlrKO and DKO mice. Thereafter, in compliance with the 3R rules for animal experiments to reduce the number of unnecessary heterozygous mice, LdlrKO and DKO strains were bred separately for the experiments. Wild-type (WT), Mmp12KO, LdlrKO, and DKO mice were housed in a clean and temperature-controlled environment (22 ± 1°C; relative humidity 45%–65%) with unlimited access to food and water on a regular 12-h light-dark cycle. At 6 weeks of age, male mice were either fed a chow diet or a high-fat, sucrose- and cholesterol-enriched diet for 13–16 weeks (HFSCD; 58 kcal% fat (primarily lard), 28 kcal% carbohydrates (with 17.5 kcal% from sucrose), 0.18% cholesterol; D09071704, Research Diets Inc). The animal experiments were carried out in accordance with the EU Directive 2010/63/EU and approved by the Federal Ministry of Science, Research, and Economy, Vienna, Austria (BMBWF-66.010/0138-V/3b/2019).

### Energy metabolism in vivo

Energy expenditure (EE), respiratory exchange ratio (RER), food and water intake, and locomotor activity were measured using automated metabolic cages (Phenomaster, TSE Systems). At the end of the respective feeding periods, LdlrKO and DKO mice were housed in the metabolic cages at 22°C for 7 days, at 5°C for 7 days (of which the middle 4 days were taken for calculations due to initial acclimation for 48 h and 12 h fasting before sacrifice) or at 5°C for 6 h. EE and RER were calculated based on indirect calorimetry data (kcal/h and VO_2_/VCO_2_) using the area under the curve (AUC) method and normalized to the 24-h measurements of the LdlrKO mice. Body temperature was measured with a rectal probe (BAT-12, Physitemp). Animals were sacrificed after 12 h of fasting.

### Plasma and BAT lipid parameters and lipoprotein analysis

Blood was collected by facial vein puncture from 12-h fasted mice. Plasma lipid parameters (TG, total cholesterol (TC)) and lipoprotein fractions were determined as previously described (25). Lipids from BAT were extracted by the Folch method and measured using enzymatic kits.

### RNA isolation and quantitative real-time PCR analysis

Total RNA was isolated using the TRISure™ reagent (Meridian, Memphis, TE) according to the manufacturer’s protocol. Two μg of total RNA were reverse transcribed to cDNA using the High-Capacity cDNA Reverse Transcription Kit (Thermo Fisher Scientific). Quantitative real-time PCR was performed on a CFX96 Real-Time PCR detection system (Bio-Rad Laboratories) using the GoTaq® qPCR Master Mix (Promega). Samples were normalized to the mRNA expression of cyclophilin A (*Ppia*) as a reference gene. Expression profiles and associated statistical parameters were determined by the 2^−ΔΔCT^ method. Primer sequences are listed in supplemental Table S1.

### Western blotting

Fifty mg of BAT were lysed in RIPA buffer supplemented with protease/phosphatase inhibitor cocktail (PIC) (1:1,000; Merck), centrifuged at 16,000 *x g* at 4°C for 30 min, the fat-free layer was isolated, and the protein concentration was determined (DC™ Protein assay, Bio-Rad Laboratories). Fifty μg of protein were separated by SDS-PAGE and blotted to a PVDF membrane. The blot was incubated with rabbit polyclonal anti-MMP12 (1:1000, # 22989-1-AP, Proteintech) and anti-calnexin (CNX) antibodies (1:1000, #2679T, Cell Signaling Technology). Monoclonal mouse anti-β-actin (ACTB) (#A5316, 1:10,000; Sigma-Aldrich) and rabbit anti-TOM20 primary antibody (#42406, 1:1,000; Cell Signaling Technology) were used as controls for the cytosolic and mitochondrial fractions, respectively. HRP-conjugated anti-rabbit (#31460, 1:2,500, Thermo Fisher Scientific, Waltham, MA) and anti-mouse (#P0260, 1:1,000, Dako) antibodies were visualized by enhanced chemiluminescence detection on a ChemiDoc™ MP imaging system (Bio-Rad Laboratories).

### Histological analyses and F4/80 immunohistochemistry

BAT was fixed in 4% neutral-buffered formalin for 24 h and embedded in paraffin. Sections (7 μm) were deparaffinized and stained with hematoxylin and eosin (H&E).

To detect macrophages, the sections were incubated with anti-mouse F4/80 antibody (1:50, #MCA497 G; Bio-Rad) for 1 h at room temperature, washed three times with PBS, incubated for 30 min with biotinylated polyclonal rabbit anti-rat immunoglobulin (1:100, #E0468; Dako, Glostrup, Denmark), and washed again thrice with PBS. To visualize the antibody binding, the sections were incubated with AEC substrate (#K3464; Dako) for 10 min. Nuclei were counterstained with Mayer’s hematoxylin for 30 s, washed with tap water, and mounted with aqueous mounting agent (#1.08562; Merck). The stained sections were visualized using an Olympus BX63 microscope equipped with an Olympus DP73 camera (Olympus).

### TOM20 immunofluorescence and confocal microscopy

The BAT sections were deparaffinized, and antigen retrieval was performed by heating the slides to 95–100°C in sodium citrate buffer (pH 6.0) for 20 min using a steamer. Immunofluorescence staining was performed as described elsewhere (26) using rabbit anti-TOM20 primary antibody (#42406, 1:200; Cell Signaling Technology) and anti-rabbit IgG Alexa Fluor™ 488 secondary antibody (#A-11008, 1:1,000; Thermo Fisher Scientific).

The stained slides were imaged on a Nikon Eclipse Ti2 inverted microscope equipped with a 100x oil (NA 1.45) objective (CFI Apochromat; Nikon), an X-Light V3 confocal/widefield module for dual color imaging (CrestOptics, Rome, Italy), and two Kinetix sCMOS cameras (Teledyne Photometrics). A 476-nm laser light (Celesta, Light Engine) with standard filters was used for excitation, and emission was collected at 501–521 nm. Three to five z-stacks of 176.8 x 176.8 μm areas were recorded at room temperature in 0.2-μm intervals per slide. Data acquisition and control of the fluorescence microscope were performed using NIS-Elements AR 5.42.06 (Nikon).

### Electron microscopy

BAT samples were fixed in 2.5% (wt/vol) glutaraldehyde/2% (wt/vol) paraformaldehyde in 0.1 M cacodylate buffer (pH 7.4) for 3 h, post-fixed in 2% (w/v) osmium tetroxide for 3 h, and dehydrated in a graded series of ethanol. The tissues were then infiltrated (overnight with propylene oxide and TAAB embedding resin, followed by 3 h incubation in pure TAAB embedding resin), transferred into embedding moulds, and polymerized (48 h, 60°C). Ultrathin sections (70 nm) were cut with a UC 7 ultramicrotome (Leica Microsystems) and stained with platinum blue for 15 min and lead citrate for 5 min. Electron micrographs were taken at 120 kV using a Tecnai G2 transmission electron microscope (Thermo Fisher Scientific) with a Gatan Ultrascan 1000 CCD camera (−20°C; Digital Micrograph acquisition software; Ametek Gatan).

### Analysis of GEO profiles database

Gene expression profiles of *Mmp12* in BAT from C57BL/6J mice were downloaded from the Gene Expression Omnibus (GEO) database repository (GSE64718; https://www.ncbi.nlm.nih.gov/geo/query/acc.cgi?acc=GSE64718; accessed on Feb 11, 2025) (27, 28, 29). We included the following groups of samples in the analysis: chow diet for 7 weeks (GSM1577825, GSM1577826, and GSM1577827), chow diet for 24 weeks (GSM1577852, GSM1577853, and GSM1577854) or a high-fat diet (HFD) for 24 weeks (GSM1577855, GSM1577856, and GSM1577857).

### Measurement of elements in plasma

An aliquot of the plasma samples (20–60 mg, weighed to the nearest 0.1 mg) was diluted with a solution containing 1% NH_4_OH, 4% 2-propanol, and 0.01% EDTA to a final volume of 5 ml. The concentrations of magnesium (Mg), phosphorus (P), iron (Fe), copper (Cu), zinc (Zn), and calcium (Ca) in the samples were determined using inductively coupled plasma mass spectrometry (ICPMS/MS) (Agilent 8,900; Agilent Technologies) at the following mass-to-charge ratios using He at a flow rate of 5 ml/min as collision gas: Mg m/z 24, Fe m/z = 56, Cu m/z = 65, Zn m/z = 66, and H_2_ as a reaction gas for Ca m/z 40. P was measured at a transition with oxygen at m/z 31 -> 47. Ge at m/z 74 serves as an internal standard. The accuracy of the concentrations was confirmed through the measurement of Clincheck plasma level I.

### Untargeted proteomic analysis of BAT

BAT samples from LdlrKO and DKO mice were processed as described elsewhere (30). The samples were analyzed using a Dionex Ultimate 3,000 nano-LC system that was connected to an Orbitrap Fusion Tribrid Mass spectrometer (Thermo Fisher Scientific) equipped with a nano-electrospray ion source. The pre-concentration of peptide mixtures, gradient elution, and MS spectra collection were previously described (31). The data acquisition was operated in the data-dependent mode (DDA) with a cycle time of 3 s between master scans. MS/MS spectra were collected in centroid mode. Higher-energy collisional dissociation was performed with a collision energy set at 35 eV. The LC-MS/MS raw files were converted to mzML format in centroid mode using the MSconvert tool software ProteoWizard (32) and then processed with an open-source, multiple peptide sequencing pipeline for label-free quantification (LFQ) based on OpenMS in the KNIME analytics platform as described (33). Peptide indexing was done against a mouse FASTA Swiss-Prot reviewed protein sequence database (uniprot-filtered-organism_Mus.musculus-(Mouse) including in the protein database a list of common contaminant proteins (n = 179, github.com/pwilmart/fasta_utilities/blob/master/Thermo_contams.fasta). Fragment mass tolerance was set at 0.02 Da and precursor mass tolerance at 5.0 ppm. Protein abundance estimates were calculated based on prior generated spectral features, followed by PIA-assisted FDR estimation and filtering at the PSM level (PSM combined FDR score > 0.01, equivalent to FDR < 1%) with subsequent filtering at the peptide and protein group level through IDfiter node options (FDR < 1%), their ID mapping and combination with peptide IDs, their subsequent grouping and normalization. The data were processed and analyzed as previously described (24). Volcano plots were generated using VolcaNoseR (https://huygens.science.uva.nl/VolcaNoseR2/) (34). Gene ontology (GO)_Biological Processes, KEGG Enrichment Analysis, and Ingenuity Pathway Analysis (IPA,QIAGEN), including canonical pathways and upstream analyses, were performed based on the differentially expressed proteins (*P* < 0.05) (35). Pathways were considered significant at an FDR <0.05. GO_Biological Processes and KEGG Enrichment Analysis were generated using String (Version 12.0), whereas for IPA data visualization was performed with R (Version 4.4.1) and the libraries used were ggplot2, dplyr, and tidyverse.

### Proteomics data analysis with MitoCarta 3.0

Mouse MitoCarta3.0 datasets related to 1140 mouse genes were downloaded from https://personal.broadinstitute.org/scalvo/MitoCarta3.0, accessed on Mar 15, 2025 (36, 37, 38). Differentially expressed proteins (*P* < 0.05) obtained from the proteomics analysis were matched with MitoCarta3.0 data using UniProtID as identifier. Overrepresented mitochondrial signaling pathways (mitopathways) were determined separately for upregulated and downregulated proteins using the Fisher exact test, comparing the proportion of proteins among all detected proteins annotated with a particular pathway with the proportion of proteins among the upregulated (downregulated) proteins annotated with that pathway. *P* values were adjusted for multiple hypothesis testing based on the FDR using the Benjamini-Hochberg method. All analyses were performed using the statistical software environment R (Version 4.3.1).

### Mitochondrial isolation and respiration analysis

Oxygen consumption rates (OCR) from isolated mitochondria were measured using a Seahorse XF Pro Analyzer (Agilent Technologies), as described previously (39). Briefly, BAT was excised, washed in cold PBS, and minced with scissors in cold MSHE buffer (mannitol (210 mM), sucrose (70 mM), HEPES (5 mM), EGTA (1 mM)) supplemented with fatty acid (FA)-free BSA (0.5% wt/vol)). The tissue was then homogenized with 8 strokes using a drill-driven Teflon dounce homogenizer (Glas-Col LLC, Terre Haute, IN). The homogenate was centrifuged at 800 *g* and 4°C for 10 min, the supernatant was collected and centrifuged again at 8,000 *g* and 4°C for 10 min. The mitochondrial pellet was washed twice with MSHE buffer to remove excess fat and resuspended in 200 μl MSHE buffer. Total protein concentration was determined using the Pierce BCA protein assay (Thermo Fisher Scientific). Mitochondria were then diluted in mitochondrial assay solution (MAS) (mannitol (220 mM), sucrose (70 mM), KH_2_PO_4_ (10 mM), MgCl_2_ (5 mM), HEPES (5 mM), EGTA (1 mM), FA-free BSA (0.2% wt/vol)) containing succinate (10 mM) as substrate, rotenone (2 μM) for complex I inhibition, and GDP (1 mM) for UCP1 inhibition so that 25 μl of solution corresponded to 2 μg of mitochondria. These were loaded into each well and centrifuged at 2,000 *x g* and 20°C for 20 min. Immediately before the measurements, 155 μl of pre-warmed MAS was added. The coupling assay was performed by sequential injection of ADP (4 mM; State 3), oligomycin (2.5 μg/ml; State 4o), FCCP (4 μM; State 3u), and antimycin A (4 μM). At baseline and after each injection, three cycles of 2-min of measurement and 2-min of mixing were performed. The OCR values of three corresponding measurement cycles were averaged and the OCR after antimycin A injection was subtracted. The resulting OCR values were normalized to the protein content.

### Mitochondrial compactness and sphericity calculations

The 3D deconvolution of the recorded z-Stacks was performed with NIS-Elements HC 5.42.01 (Nikon). The background and autofluorescence were subtracted from the images. At least two areas of 32.5 × 32.5 μm were selected from each image for further analysis. The 3D sphericity and compactness were then estimated using ImageJ2 (40) and the 3D RoiManager plugin (41). In short, Otsu’s automatic thresholding algorithm was applied to the images to identify 3D objects. The compactness was calculated as a normalized ratio between the surface and the volume of a mitochondrion, whereas sphericity was defined as the cube root of compactness.

### Statistical analyses

Statistical analyses were performed using GraphPad Prism (Version 9.3.1) software and R (Version 4.4.1). Data are presented as mean ± SD. Significances were calculated using the unpaired Student’s *t* test or one-way ANOVA followed by Bonferroni post hoc tests. The following levels of statistical significance were used: ∗*P* < 0.05, ∗∗*P* ≤ 0.01, ∗∗∗*P* ≤ 0.001.

## Results

### Improved metabolic activity and reduced TG concentrations in BAT from HFSCD-fed DKO mice

**Fig. 1:**
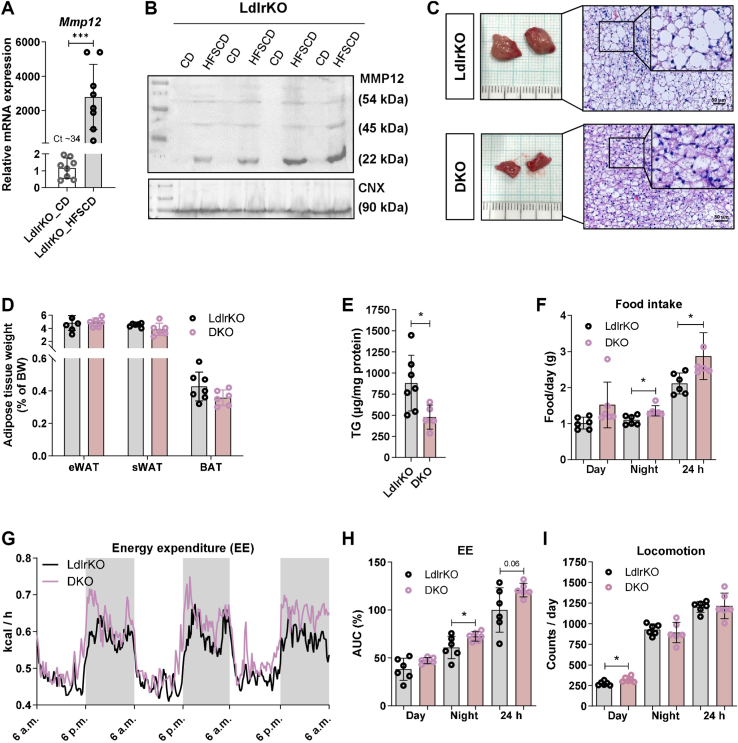
*Mmp12*/MMP12 is upregulated in BAT upon HFSCD feeding and its loss is associated with reduced BAT triglyceride content and improved energy expenditure. Male LdlrKO mice were sacrificed at the age of 23 weeks (chow diet (CD)) or 20–26 weeks after 13–16 weeks of high-fat, sucrose- and cholesterol-enriched diet (HFSCD) feeding. A, *Mmp12* mRNA expression in BAT from LdlrKO mice relative to *Ppia* expression (n = 8). B, MMP12 protein abundance (54-kDa inactive precursor, 45-kDa intermediate, and fully active 22-kDa from) in BAT from LdlrKO mice with Calnexin (CNX) as loading control (n = 4). C, macroscopic images and H&E staining of BAT sections (scale bar, 50 μm). D, weight of epididymal (eWAT) and subcutaneous (sWAT) white adipose tissue as well as BAT as percentage of body weight. *E*, triglyceride (TG) concentrations in the BAT of HFSCD-fed LdlrKO and DKO mice (n = 5–7). F, daily food intake, (G) energy expenditure (EE), and (H) the corresponding calculation for the area under the curve (AUC). I, daily locomotor activity of HFSCD-fed mice (n = 6). Data represent mean values ± SD. Statistical differences were calculated with Student’s *t* test; ∗*P* < 0.05, ∗∗∗*P* ≤ 0.001.

*Mmp12* gene (Fig. 1A; Ct value of 34.6) and the expression of the fully active 22-kDa form of the MMP12 protein (Fig. 1B) were absent in BAT isolated of chow diet-fed LdlrKO mice. Consistent with this finding, *Mmp12* mRNA expression was absent in primary brown adipocytes of LdlrKO and WT mice (supplemental Fig. S1A; Ct values > 35). mRNA and protein expression were massively increased in BAT after 16 weeks of HFSCD feeding (Fig. 1A, B). We found a similar *Mmp12* upregulation in C57BL/6J mice fed with a high-fat diet for 24 weeks in comparison with chow diet-fed mice in publicly available datasets (27) (supplemental Fig. S1B). Genetic deletion of *Mmp12* in 16-weeks HFSCD-fed LdlrKO mice was associated with slightly reduced body weight (24) and decreases in BAT size (Fig. 1C), mass (Fig. 1D), and TG content (Fig. 1E), indicating a potential role of MMP12 in BAT function in this cardiometabolic mouse model. These observations were also reflected in Mmp12KO and WT mice after 16 weeks of HFSCD feeding (supplemental Fig. S2A, B). Consistent with elevated food intake (Fig. 1F) and the observed changes in BAT, DKO mice showed increased EE (Fig. 1G, H). Of note, we used absolute EE values without taking body weight into account, as our system does not allow continuous measurement of body weight, thus preventing reliable data for ANCOVA and CalR determinations. Locomotion of DKO mice was slightly elevated during the day but unaltered during the active phase (night) (Fig. 1I). Collectively, these observations indicate that genetic loss of *Mmp12* may improve HFSCD-induced BAT dysfunction, concomitant with beneficial adaptations in systemic energy expenditure.

### Altered proteome signature and improved metabolic function in BAT from HFSCD-fed DKO mice

**Fig. 2:**
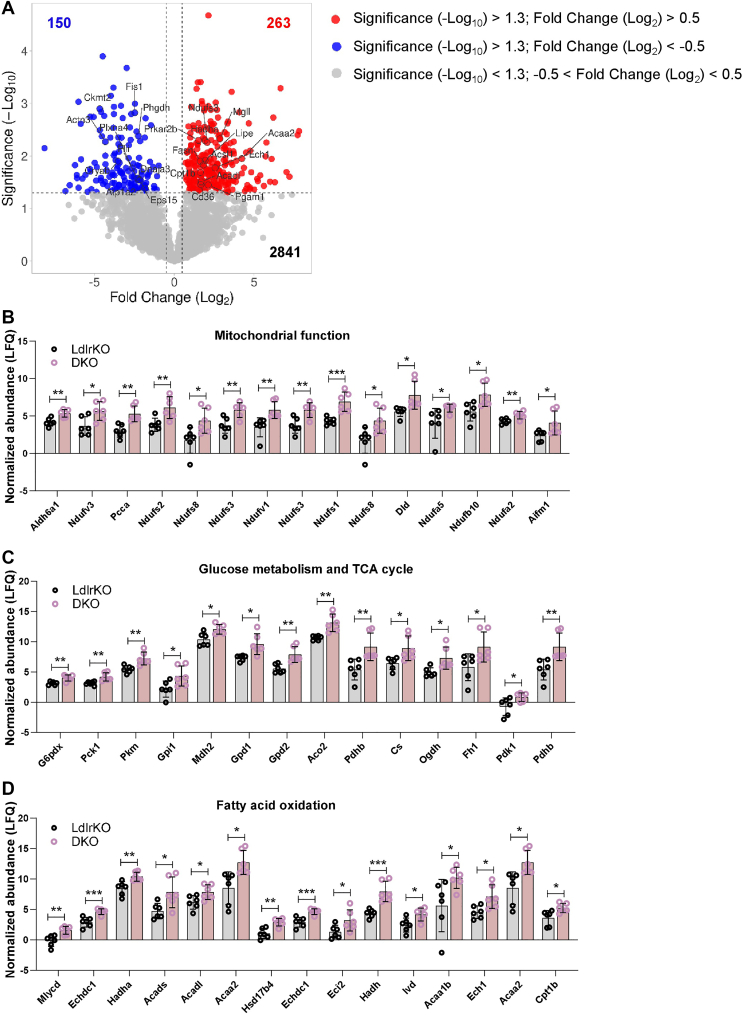
Altered proteome signature in the BAT of DKO mice. Male LdlrKO and Ldlr/Mmp12 DKO mice were fed HFSCD for 13–16 weeks and BAT was isolated after 12 h of fasting. A, Volcano plot showing significantly upregulated (red) and downregulated proteins (blue) in the BAT from DKO mice. Normalized label-free quantification (LFQ) abundance of proteins related to (B) mitochondrial function, (C) glucose metabolism and TCA cycle, and (D) fatty acid oxidation in the BAT of both genotypes. Data represent mean values (n = 6) ± SD; ∗*P* < 0.05, ∗∗*P* ≤ 0.01, ∗∗∗*P* ≤ 0.001.

To delineate the pathways affected by MMP12 deficiency in BAT, we performed untargeted proteomics from BAT of HFSCD-fed LdlrKO and DKO mice. Out of 3,254 label-free quantified proteins with no missing values, we identified 413 with differential abundance in BAT from DKO mice (*P* < 0.05), with 150 being downregulated (Log_2_FC < −0.5) and 263 being upregulated (Log_2_FC > 0.5) (Fig. 2A and supplemental File 2). Among the 150 downregulated proteins, we found FIS1, CKTM2, ACTN3, PLXNA4, FLII, DNAJA3, PHGDH, CRYAB, ATP1A2 and EPS15, whereas NDUFS3, MGLL, PRKAR2B, HADHA, ACSL2, ECH1, ACADL, PGAM1, CD36, CPT1B, FASN, LIPE and ACAA2 were upregulated in the DKO BAT (Fig. 2A, supplemental File 2 and supplemental Table S1). According to the PubMed Advanced Search Builder tool, all of these proteins were associated with the search terms “thermogenesis” and/or “brown adipose tissue”. Consistent with these results, the analysis also revealed numerous proteins related to mitochondrial function, glucose metabolism and tricarboxylic acid (TCA) cycle, and fatty acid (FA) oxidation being upregulated in the BAT from DKO mice (Fig. 2B–D).

**Fig. 3:**
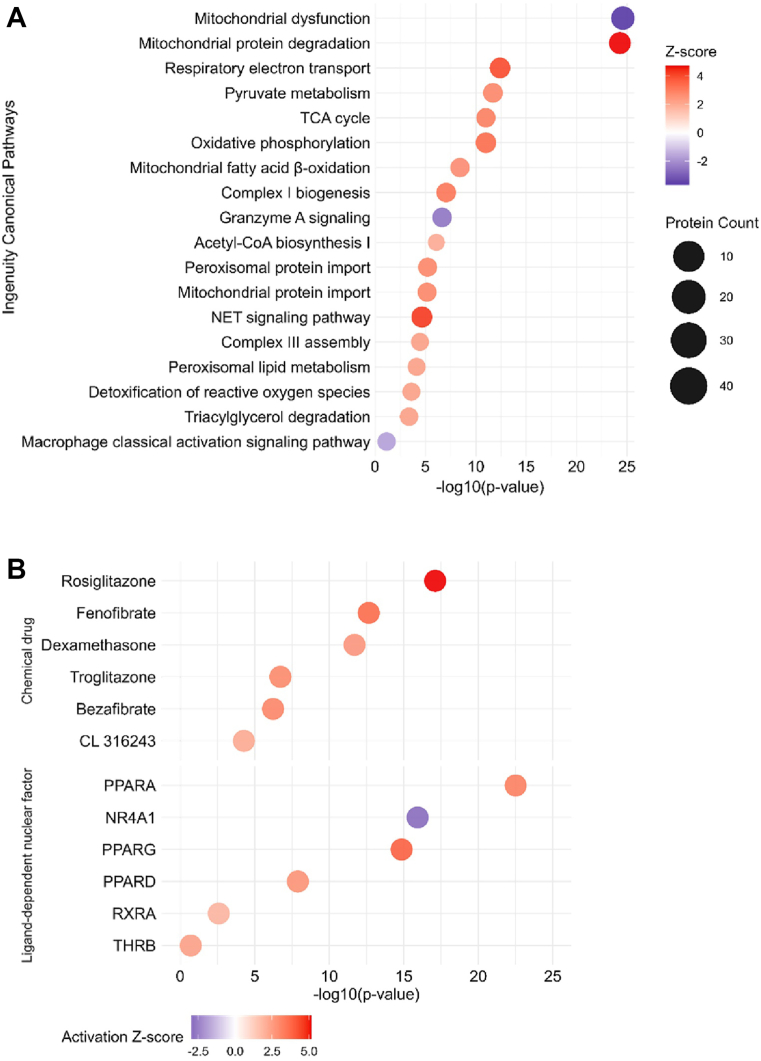
Dysregulation of mitochondria- and inflammation-related pathways and thermogenesis-associated upstream regulators in DKO BAT. Male LdlrKO and Ldlr/Mmp12 DKO mice (n = 6) were fed HFSCD for 13–16 weeks and BAT was isolated after 12 h of fasting. Ingenuity Pathway Analysis (IPA) from untargeted proteomics of BAT showing (A) canonical pathways and upstream analysis of (B) chemical drugs and ligand-dependent nuclear factors dysregulated in DKO BAT. Pathways and upstream regulators predicted to be inhibited in BAT of DKO mice are shown in blue (z-score < -2), pathways and upstream regulators predicted to be activated in DKO mice are shown in red (z-score >2).

Next, we performed pathway analyses to evaluate whether the changes observed at the proteome level led to variations in the regulation of molecular processes and functions. Based on the analyses of KEGG pathways and Gene Ontology Biological Processes, we noticed the enrichment of metabolic pathways, carbon, pyruvate, small molecule, amino acid and FA metabolism/degradation, glycolysis and gluconeogenesis, TCA cycle, and cellular respiration among others, in the DKO BAT (supplemental Fig. S3A, B, supplemental File S2 and supplemental Tables S2 and S3). To predict whether the enriched pathways identified were likely activated or inhibited, we employed the Ingenuity Pathway Analysis (IPA) revealing that mitochondrial protein degradation, respiratory electron transport, pyruvate metabolism, TCA cycle, oxidative phosphorylation, and FA β-oxidation were among the upregulated canonical pathways (Fig. 3A, supplemental File S2, and supplemental Table S4). In line with this, mitochondrial dysfunction was the most downregulated pathway, indicating that mitochondrial function is improved by MMP12 loss. To gain mechanistic insights and anticipate whether a pathway in the DKO BAT is affected in a manner comparable to the effect of a drug, we employed the Upstream Regulators section in IPA to predict other molecules or transcription factors that might be responsible for the observed changes in protein abundance. The activation of rosiglitazone, fenofibrate, dexamethasone, troglitazone, bezafibrate, and CL 316243 (Fig. 3B, supplemental File S2, and supplemental Table S5) indicate that these chemical drugs induce a protein expression pattern similar to that observed in the DKO BAT and reminiscent of the known responses to these compounds. In addition, we identified PPARA, PPARG, PPARD, and RXRA as predicted activated ligand-dependent nuclear receptors (Fig. 3B, supplemental File S2, and supplemental Table S5), suggesting that the same downstream proteins are being targeted in the DKO BAT. We also noted an activation of PPARGC1A (PGC-1α) and the browning hormone FGF21 as well as an inhibition of miR-433-3p and miR-494-3p, negative regulators of thermogenesis and mitochondrial biogenesis through PGC-1α (42, 43) (supplemental File S2 and supplemental Table S5).

Taken together, these data suggest that the deletion of MMP12 in HFSCD-fed LdlrKO mice alters the expression signature of numerous proteins, pathways, and regulators in the BAT toward a more thermogenically active metabolic phenotype.

### Reduced macrophage infiltration and expression of lipid-associated macrophage markers in BAT from DKO mice

**Fig. 4:**
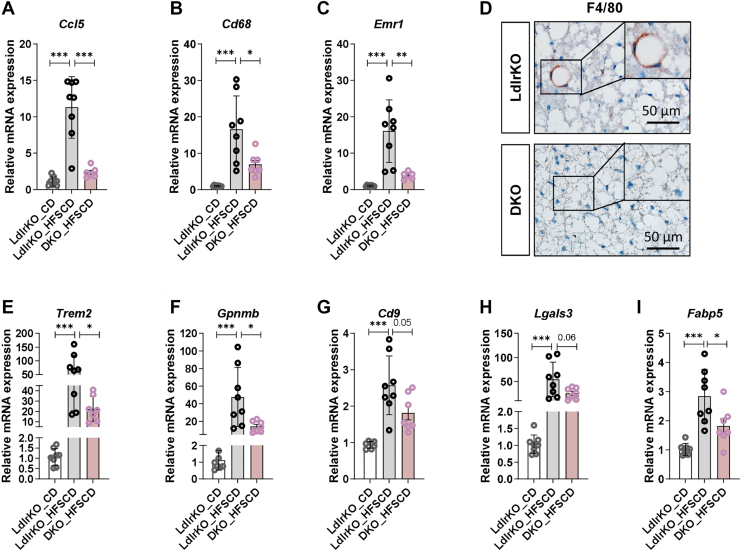
Decreased macrophage infiltration and reduced expression of lipid-associated macrophage markers in DKO BAT. BAT was isolated after 12 h of fasting from age-matched male CD-fed LdlrKO as well as LdlrKO and Ldlr/Mmp12 DKO mice fed HFSCD for 13–16 weeks mRNA expression of the macrophage markers (A) *Ccl5*, (B) *Cd68*, and (C) *Emr1* relative to *Ppia* expression. (D) F4/80 immunohistochemistry in BAT sections (scale bar, 50 μm). mRNA expression of the lipid-associated macrophage markers (E) *Trem2*, (F) *Gpnmb*, (G) *Cd9*, (H) *Lgals3*, and (I) *Fabp5* relative to *Ppia* expression. Data represent mean values (n = 6–8) ± SD. Statistical differences were calculated with one-way ANOVA followed by Bonferroni post-hoc tests; ∗*P* < 0.05, ∗∗*P* ≤ 0.01, ∗∗∗*P* ≤ 0.001.

IPA also revealed the downregulation of inflammation-related pathways in DKO BAT, including granzyme A signaling and macrophage classical activation signaling (Fig. 3A). We therefore analyzed mRNA expression of macrophage marker genes in BAT from LdlrKO and DKO mice and included BAT from chow diet-fed LdlrKO mice as control. Compared to chow-fed LdlrKO mice, we observed a 12-fold upregulation of the macrophage chemoattractant *Ccl5*, which was markedly reduced in DKO mice (Fig. 4A). Consistent with this result, the 17- and 16-fold increase in the expression of the macrophage markers *Cd68* and *Emr1* in LdlrKO BAT upon HFSCD-feeding were reduced by more than 50% in DKO BAT (Fig. 4B, C), indicating attenuated infiltration of macrophages into the BAT of DKO mice. The decreased abundance of *Emr1* was further confirmed by F4/80 immunohistochemistry (Fig. 4D). Furthermore, the LAM markers *Trem2*, *Gpnmb*, *Cd9*, *Lgals3*, and *Fabp5*, which were also upregulated in LdlrKO mice by HFSCD feeding, were reduced in the BAT from DKO mice (Fig. 4E–I). These results support the proteomics data and further suggest that the loss of MMP12 decreases macrophage infiltration and the gene expression signature of LAMs in BAT upon HFSCD feeding, which could ultimately contribute to an improved metabolic homeostasis of the tissue.

### MMP12 expression in the mitochondrial fraction of BAT and reduced oxygen consumption, compactness, and sphericity of mitochondria in BAT from DKO mice

**Fig. 5:**
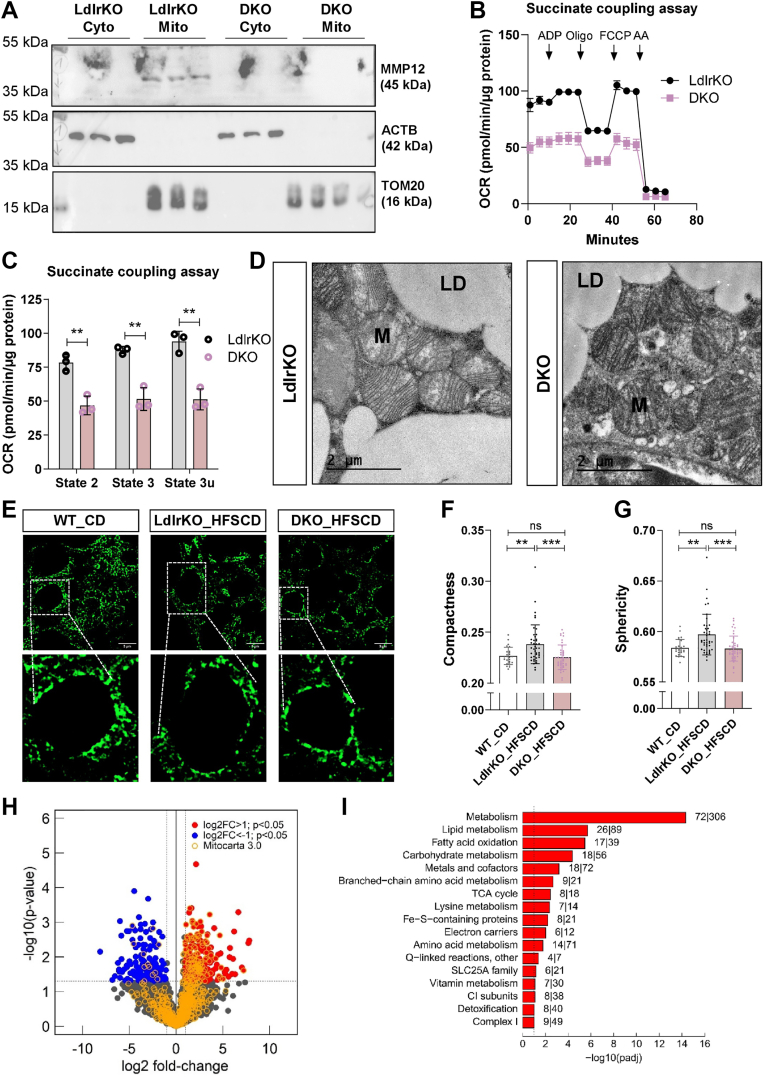
MMP12 expression in the mitochondrial fraction of BAT and reduced oxygen consumption, compactness, and sphericity of mitochondria in DKO BAT. Male LdlrKO and Ldlr/Mmp12 DKO mice were fed HFSCD for 13–16 weeks and BAT was isolated after 12 h of fasting. *A*, MMP12 protein abundance in cytosolic (Cyto) and mitochondrial (Mito) fractions of BAT. β-actin (ACTB) and TOM20 were used as controls for the cytosolic and mitochondrial fractions, respectively. B, Representative oxygen consumption rates (OCR) from isolated BAT mitochondria respiring with succinate as energy substrate and sequentially treated with ADP, oligomycin (Oligo), carbonyl cyanide-p-trifluoromethoxyphenylhydrazon (FCCP), and antimycin A (AA) injections. C, quantification of OCR at basal condition (State 2) and after injection of ADP (State 3) and FCCP (State 3u) (n = 3). D, Electron micrographs of BAT sections from LdlrKO and DKO mice showing lipid droplets (LD) and mitochondria (M) (scale bar, 2 μm). E, representative images of TOM20 immunofluorescence staining in BAT sections of age-matched CD-fed WT, HFSCD-fed LdlrKO and Ldlr/Mmp12 DKO mice (scale bar. 5 μm). F, mitochondrial compactness and (G) sphericity calculated from TOM20 immunofluorescence staining (n = 25–41). H, Volcano plot after MitoCarta 3.0 analysis showing upregulated (red), downregulated (blue), and mitochondrial (yellow) proteins in the BAT from DKO mice. I, upregulated mitochondrial pathways in DKO BAT obtained by MitoCarta 3.0 analysis. The numbers next to each bar represent the number of proteins detected in the proteomics analysis over the total number of mitochondrial proteins annotated in each pathway according to MitoCarta 3.0. Data represent mean values (n = 6) ± SD; ∗∗*P* ≤ 0.01, ∗∗∗*P* ≤ 0.001.

Among the most affected pathways in DKO BAT identified by proteomics and pathway analyses were mitochondrial-related proteins and pathways (Figs. 2B and 3A), indicating that the deletion of MMP12 may also directly ameliorate mitochondrial function. We therefore determined whether MMP12 may also be present in the mitochondrion to eventually regulate its function, comparable to the described function of MMP12 as a transcription factor after its translocation to the nucleus (18). Using Western blot analysis after BAT fractionation, we detected the expression of MMP12 in the mitochondrial fraction, as confirmed by TOM20 expression as positive control (Fig. 5A). Based on the absence of *Mmp12* gene expression in primary brown adipocytes, MMP12 is rather secreted by macrophages, taken up by brown adipocytes, and translocated to mitochondria. To elucidate whether MMP12 modulates mitochondrial function independently of any other cellular effect, we isolated mitochondria from BAT and performed mitochondrial respirometry. Interestingly, we observed reduced oxygen consumption rate (OCR) in isolated mitochondria from the DKO mice (Fig. 5B, C), despite comparable mRNA expression of the mitochondrial biogenesis markers *16S* and *Nd1* (supplemental Fig. S4A) and ultrastructure (matrix and cristae architecture) between BAT sections from both genotypes (Fig. 5D and supplemental Fig. S4B, C). Since mitochondria often appear rounded in electron micrographs due to the sectioning plane and does not necessarily reflect their dynamic and variable shapes, we next stained BAT sections with TOM20 to visualize the mitochondria around the lipid droplets (Fig. 5E). Analysis after 3D deconvolution of the confocal fluorescence micrographs revealed that DKO tissues had reduced mitochondrial compactness and sphericity, comparable to chow-fed WT mice that were analyzed as a control (Fig. 5F, G).

To get a deeper understanding of specific mitochondrial processes and functions driven by the differences in respiration observed between both genotypes, we reanalyzed our BAT proteomics data from LdlrKO and DKO mice with MitoCarta 3.0 (38), which contains a curated and high confidence inventory of mitochondrial proteins. Consistent with our observations, we identified 105 upregulated and only 11 downregulated mitochondrial proteins in the DKO BAT (Fig. 5H). The MitoCarta analysis revealed upregulation of additional mitochondrial-related pathways, including branched-chain amino acid metabolism, lysine metabolism, electron carriers, amino acid metabolism, Q-linked reactions, SLC25A family, vitamin metabolism, CI subunits, detoxification, and complex I (Fig. 5I). In addition, Fe-S-containing proteins were increased in DKO BAT (Fig. 5I), consistent with the notion that brown adipocytes rely on iron-depending mitochondrial enzymes for proper function. In agreement, we observed a trend of increase in circulating iron concentrations in DKO mice (supplemental Fig. S5).

Together, these findings suggest that MMP12 localizes also in the mitochondrial fraction in BAT and that its deficiency is associated with altered respiration, sphericity, and compactness of mitochondria and increased expression of mitochondrial proteins and pathways.

### Increased metabolic activity with unchanged plasma lipid parameters and thermogenic gene expression in BAT from HFSCD-fed DKO mice after 7 days of cold exposure

**Fig. 6:**
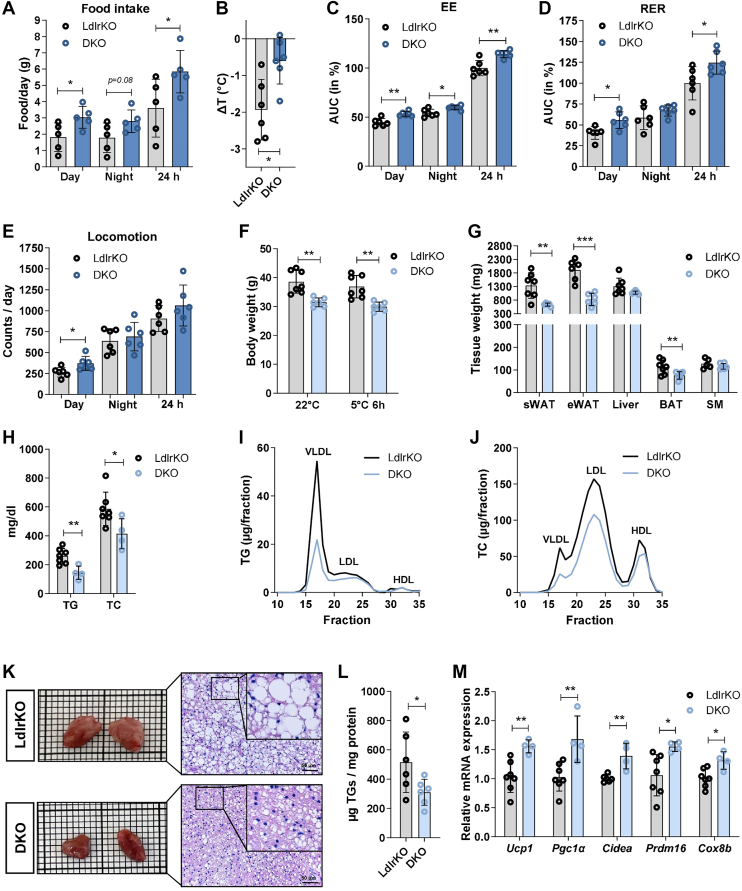
Reduced circulating lipid concentrations and increased BAT function in HFSCD-fed DKO mice after 6 h of cold exposure. Male LdlrKO and Ldlr/Mmp12 DKO mice were fed HFSCD for 13–16 weeks and exposed to 5°C for (A–E) the last 7 days or (F-M) for 6 h and fasted for 12 h before sacrifice. A, Daily food intake (n = 5; one mouse per genotype did not eat properly, so we placed the pellets on the floor of the two cages) and (B) body temperature differences between before and after 7 days of cold exposure (n = 6). Area under the curve (AUC) calculated from (C) energy expenditure (EE) and (D) respiratory exchange ratio (RER) during the middle days of 7 days of cold exposure (n = 6). E, daily locomotor activity (n = 6). F, body weight at 22°C and after cold exposure at 5°C for 6 h (n = 6–7). G, tissue weights (n = 5–7), (H) plasma triglyceride (TG), total cholesterol (TC) (n = 5–7), and lipoprotein profiles for (I) TG and (J) TC in pooled plasma samples after 6-h cold exposure after fast protein liquid chromatography separation (n = 4–7). K, Macroscopic images and H&E staining of BAT sections (scale bar, 50 μm). L, TG concentrations (n = 6) and (M) mRNA expression of browning markers in BAT relative to *Ppia* expression (n = 4–7). Data represent mean values ± SD. Statistical differences were calculated with Student’s *t* test; ∗*P* < 0.05, ∗∗*P* ≤ 0.01, ∗∗∗*P* ≤ 0.001.

Since our results so far have shown that BAT from DKO mice exhibits an improved metabolic function, we exposed HFSCD-fed mice to 5°C for 7 days. Despite increased food intake (Fig. 6A), we observed comparable body weights (supplemental Fig. S6A), tissue weights except for a slight raise in epididymal WAT (eWAT) weight in the DKO mice (supplemental Fig. S6B) and plasma lipid parameters (supplemental Fig. S6C) between both genotypes. However, DKO mice showed improved tolerance to environmental temperature drop (Fig. 6B) as well as an even more pronounced increase in EE, RER, and daily locomotion than at 22°C (Fig. 6C–E, supplemental Fig. S6D, E). Despite these differences, BAT from LdlrKO and DKO mice had comparable size, histology, TG content, and mRNA expression of thermogenic genes, except for a minor increase in *Ucp1* (supplemental Fig. S6F–H). We therefore speculated that acclimation to cold exposure during 7 days may have been such a potent trigger of BAT activation in LdlrKO mice, leading to a TG pool depletion and thereby masking innate BAT activation in DKO mice.

### Improved plasma lipid parameters and BAT function from HFSCD-fed DKO mice after 6 h of cold exposure

To test this possibility and capture earlier genotype-dependent BAT adaptations prior to complete cold acclimation, we next investigated the metabolic and molecular effects of 6-h cold exposure. This short-term cold was associated with lower body weight and weights of subcutaneous WAT (sWAT), eWAT, and BAT in DKO compared to Ldlr KO mice (Fig. 6F, G). In agreement with the improved cardiometabolic phenotype and reduced circulating lipid parameters observed in DKO mice housed at 22°C (24), acute cold-exposed DKO mice had also significantly decreased circulating TG and TC concentrations (Fig. 6H), particularly in apolipoprotein B-containing lipoproteins and the VLDL-TG fraction (Fig. 6I, J). The BAT size was also markedly decreased and the color was darker compared to the light brown LdlrKO BAT (Fig. 6K). H&E staining of BAT sections showed much smaller lipid droplets in DKO versus LdlrKO BAT (Fig. 6K), which was confirmed by a less pronounced TG accumulation (Fig. 6L). In line, mRNA expression levels of the thermogenic genes *Ucp1*, *Pgc1α*, *Cidea*, *Prdm16*, and *Cox8b* were increased (Fig. 6M). These data indicate that the numerous positive effects of MMP12 deficiency on whole-body energy metabolism as well as BAT size and TG content at RT can be further enhanced by acute cold exposure.

## Discussion

BAT is a central metabolic organ that plays a key role in the regulation of energy expenditure by burning calories in the form of heat. Beyond its well-known thermogenic capacity, BAT activation offers numerous benefits for systemic health, particularly by triggering TG turnover and the release of batokines (3, 4, 5, 6). Therefore, molecular targets that promote BAT function are a highly attractive avenue for the development of therapeutic strategies to combat obesity-associated metabolic disorders. In this study, we demonstrated that the constitutive loss of MMP12 exerts beneficial effects on whole-body energy homeostasis, acute cold-induced BAT function, mitochondria-related processes in BAT, macrophage infiltration, and inflammation, although in most but not all of our cohorts, body weights at 22°C were unchanged or reduced (24). This discrepancy might (at least in part) depend on whether the mice were fed a HFSCD for 13, 15, or 16 weeks. MMPs have traditionally been associated with ECM remodeling-associated functions. However, many members of the MMP family, such as MMP3, 7, 9, 10, 14, and 26 exert proteolytic functions in various intracellular compartments (44, 45, 46, 47, 48, 49). In contrast, MMP14 also localized in the intracellular compartment, serves proteolytic-independent functions and regulates energy production in macrophages by activating hypoxia-inducible factor-1 via a non-proteolytic mechanism (50). MMP12 has been reported to act as a transcription factor in the nucleus of virus-infected cells (18) and induce bacterial cell death in phagolysosomes of macrophages (19). In mice, circulating MMP12 is primarily abundant in its ∼45-kDa form and the 22 kDa catalytic fragment is absent because activation and fragmentation occur locally in tissues, not in the circulation, where it would be rapidly inactivated or degraded. Although soluble MMP12 was shown to bind cellular membranes followed by internalization (17), the mechanism by which MMP12 may enter the mitochondria remains elusive. The appearance of MMP12 in the mitochondrial fraction, however, could also be due to colocalization. The mitochondria in BAT might surround other organelles such as lipid droplets, so MMP12 could be located at contact points between the organelles and not necessarily within the mitochondria.

In agreement with our findings in BAT, a previous study reported that macrophage-produced MMP12 targets white adipocytes, leading to mitochondrial damage, inflammation, and insulin resistance (22). The authors also showed that the treatment of high-fat, high-sugar diet-fed mice with a small molecule MMP12 inhibitor restored mitochondria-related pathways, as expression of mitochondrial genes was increased and oxidative stress markers were decreased in WAT (22). In addition to the changes in DKO mice in terms of EE, BAT phenotype, BAT proteome signature, and activation of metabolic pathways, we observed that the compactness and sphericity of mitochondria were comparable to the shape in BAT of chow-fed LdlrKO mice, indicating improved mitochondrial shape change in DKO BAT. In isolated mitochondria from the BAT of DKO mice, however, we measured reduced OCR. At first glance, this finding looks like a discrepancy, as reduced OCR generally goes hand in hand with diminished mitochondrial function. However, spherical and highly compact mitochondria are an indication of exposure to reactive oxygen species (ROS) and might explain the increased OCR in HFSCD-fed LdlrKO cells. Consistent with these findings, BAT of aged C57BL/6J mice fed a high-fat or high-sucrose diet also exhibited an increased basal and maximal OCR compared to young mice fed chow diet (51). Similar results were also observed in humans, where adipocytes from metabolically unhealthy obese individuals showed higher mitochondrial respiration than those from healthy obese individuals (52). Mitochondrial ROS production plays a pivotal role in the development of age-related disorders, including cardiometabolic diseases (53), as mitochondrial oxygen consumption diverts to ROS production to a much greater extent during pathological than physiological states (54). Several reasons for enhanced mitochondrial oxygen consumption may lead to ROS production and impairment of either the electron transport chain and/or proton leakage. According to our MitoCarta-generated data, these pathological effects are likely improved in the DKO mice, indicating that the BAT from DKO mice may have the capacity to utilize oxygen more efficiently with less ROS production, likely explaining the reduced OCR in the isolated mitochondria from DKO mice. Thus, the reduced compactness and sphericity of the mitochondria in the DKO BAT, which resembled the phenotype of chow-fed WT mice, further indicated that MMP12 deficiency may ameliorate the mitochondrial phenotype in cardiometabolic diseases.

The dynamics of immune cells, especially of all the different macrophage populations, have traditionally focused on WAT function. In recent years, however, a growing body of evidence has suggested that these cells are also critical players in BAT physiology. Based on undetectable *Mmp*12 mRNA and protein expression in BAT of chow-diet fed LdlrKO mice, its absence in iBACs (Ct > 33), primary brown adipocytes of LdlrKO and WT mice shown in this study as well as in chow-diet fed Wt mice (55), we concluded that MMP12 expression in BAT originates from macrophages, whose increased occurrence is caused by HFSCD feeding. Consistent with this, the number of macrophages increases in tissues, including BAT, during the development of various diseases and is associated with the increase in MMP12 expression (11, 12, 13, 24). Its lack in chow diet conditions indicate the absence of expression in brown adipocytes, suggesting that mice with global instead of BAT-specific MMP12 deficiency may not exhibit an altered phenotype.

In pathological states, such as after vascular injury, cardiovascular disease, type 2 diabetes, fibrotic disorders, or systemic inflammation, humans present increased serum/plasma levels of MMP12 (56, 57). We have also detected a 1.4-fold higher concentration of circulating MMP12 in sera from patients with metabolic syndrome compared to healthy volunteers (24). Circulating MMP12 levels, however, are generally much lower than in tissues, most probably because circulating monocytes upregulate MMP12 only after differentiation or activation into macrophages (by e.g. GM-CSF, M-CSF, or LPS/IFNγ stimulation). Based on the observed consequences of HFSCD feeding on BAT shown in this study and on other tissues such as eWAT and aorta (24), the specific role of macrophage-secreted MMP12 on BAT function might be elucidated by performing bone marrow transplantation in the future.

Several studies have detailed how BAT-resident macrophages participate in the mitochondrial quality control system by removing extracellular mitochondrial waste to support the thermogenic function of metabolically stressed adipocytes (58). In line, infiltrating monocytes were shown to differentiate into anti-inflammatory macrophages as a response to efferocytosis of these mitochondrial-derived vesicles (58). As macrophage depletion in mice resulted in thermogenic impairment of BAT and inguinal WAT (59, 60), an absence of these macrophages could represent an additional pathogenic contributor to a metabolic imbalance (60). The reduced abundance of inflammatory macrophages and the diminished expression of proteins associated with inflammation-related pathways in BAT is therefore consistent with the improved function of the DKO BAT. Based on only a slight upregulation of FASN but no other proteins involved in lipogenesis, the data also indicate that the observed phenotype is likely due to increased FA oxidation rather than decreased de novo lipogenesis. Alterations in the gene expression signature of LAMs in the DKO BAT are in line with the finding that LAMs are a driver of loss of brown adipocyte fingerprint in both genetic and diet-induced obesity (21). LAMs scavenge extracellular vesicles loaded with damaged lipids and mitochondria that are released from brown adipocytes. However, they also release TGF-β1, which is responsible for the loss of brown adipocyte identity and the development of foam cell-like characteristics due to high expression of genes related to lipid handling and phagocytosis (21). MMP12 deficiency is associated with changes in the expression of several of these genes. However, further research is needed to clarify to which extent the loss of MMP12 directly affects the function of LAMs. Finally, the positive effects of MMP12 deficiency on BAT size and TG content were further enhanced by acute cold exposure. The observed metabolic improvements during acute cold exposure, including elevated energy expenditure and improved circulating lipid parameters, might also be the consequences of a beneficial impact of muscle shivering. Although we cannot rule out the that the thermogenic response is primarily driven by muscle shivering rather than by BAT activity (61), our results demonstrate the beneficial effect of MMP12 loss on whole-body and BAT metabolism.

We conclude that besides its beneficial effects on insulin sensitivity, systemic inflammation, and atherosclerosis, the deletion of MMP12 improves brown adipose tissue function in cardiometabolic conditions. Given that MMP12 is a secreted protein, the consequences of its pharmacological inhibition should be investigated in future studies. If successful, MMP12 inhibition in cardiometabolic mice may form the basis for therapeutic treatments of cardiometabolic diseases. This in turn could lead to our findings being translated into therapeutic applications in humans.

### Data availability

The mass spectrometry datasets have been deposited to the ProteomeXchange Consortium via the PRIDE repository (62), with the dataset identifier PXD063623.

### Supplementary data

This article contains supplemental data.

## Conflict of interest

The authors declare that they have no conflicts of interest with the contents of this article.

## References

[1] Rosen E.D., Spiegelman B.M. (2014). What we talk about when we talk about fat. Cell.

[2] Saito M., Matsushita M., Yoneshiro T., Okamatsu-Ogura Y. (2020). Brown adipose tissue, diet-induced thermogenesis, and thermogenic food ingredients: from mice to men. Front. Endocrinol..

[3] Wang G.X., Zhao X.Y., Lin J.D. (2015). The brown fat secretome: metabolic functions beyond thermogenesis. Trends Endocrinol. Metab..

[4] Villarroya F., Cereijo R., Villarroya J., Giralt M. (2017). Brown adipose tissue as a secretory organ. Nat. Rev. Endocrinol..

[5] Bartelt A., Bruns O.T., Reimer R., Hohenberg H., Ittrich H., Peldschus K. (2011). Brown adipose tissue activity controls triglyceride clearance. Nat. Med..

[6] Verkerke A.R.P., Wang D., Yoshida N., Taxin Z.H., Shi X., Zheng S. (2024). BCAA-nitrogen flux in brown fat controls metabolic health independent of thermogenesis. Cell.

[7] Becher T., Palanisamy S., Kramer D.J., Eljalby M., Marx S.J., Wibmer A.G. (2021). Brown adipose tissue is associated with cardiometabolic health. Nat. Med..

[8] Morrison C.J., Butler G.S., Rodriguez D., Overall C.M. (2009). Matrix metalloproteinase proteomics: substrates, targets, and therapy. Curr. Opin. Cell Biol..

[9] Löffek S., Schilling O., Franzke C.W. (2011). Series "matrix metalloproteinases in lung health and disease": biological role of matrix metalloproteinases: a critical balance. Eur. Respir. J..

[10] Jobin P.G., Butler G.S., Overall C.M. (2017). New intracellular activities of matrix metalloproteinases shine in the moonlight. Biochim. Biophys. Acta Mol. Cel. Res..

[11] Jiang L., Yang M., He S., Li Z., Li H., Niu T. (2021). MMP12 knockout prevents weight and muscle loss in tumor-bearing mice. BMC Cancer.

[12] Lagente V., Le Quement C., Boichot E. (2009). Macrophage metalloelastase (MMP-12) as a target for inflammatory respiratory diseases. Expert Opin. Ther. Targets.

[13] Chelluboina B., Nalamolu K.R., Klopfenstein J.D., Pinson D.M., Wang D.Z., Vemuganti R. (2018). MMP-12, a promising therapeutic target for neurological diseases. Mol. Neurobiol..

[14] Guan C., Xiao Y., Li K., Wang T., Liang Y., Liao G. (2019). MMP-12 regulates proliferation of mouse macrophages via the ERK/P38 MAPK pathways during inflammation. Exp. Cell Res..

[15] Nénan S., Planquois J.M., Berna P., De Mendez I., Hitier S., Shapiro S.D. (2005). Analysis of the inflammatory response induced by rhMMP-12 catalytic domain instilled in mouse airways. Int. Immunopharmacol..

[16] Yang M., Zhang X., Liu Q., Niu T., Jiang L., Li H. (2020). Knocking out matrix metalloproteinase 12 causes the accumulation of M2 macrophages in intestinal tumor microenvironment of mice. Cancer Immunol. Immunother..

[17] Koppisetti R.K., Fulcher Y.G., Jurkevich A., Prior S.H., Xu J., Lenoir M. (2014). Ambidextrous binding of cell and membrane bilayers by soluble matrix metalloproteinase-12. Nat. Commun..

[18] Marchant D.J., Bellac C.L., Moraes T.J., Wadsworth S.J., Dufour A., Butler G.S. (2014). A new transcriptional role for matrix metalloproteinase-12 in antiviral immunity. Nat. Med..

[19] Houghton A.M., Hartzell W.O., Robbins C.S., Gomis-Ruth F.X., Shapiro S.D. (2009). Macrophage elastase kills bacteria within murine macrophages. Nature.

[20] Xu R., Vujic N., Bianco V., Reinisch I., Kratky D., Krstic J. (2024). Lipid-associated macrophages between aggravation and alleviation of metabolic diseases. Trends Endocrinol. Metab..

[21] Sciarretta F., Ninni A., Zaccaria F., Chiurchiu V., Bertola A., Karlinsey K. (2024). Lipid-associated macrophages reshape BAT cell identity in obesity. Cell Rep.

[22] Li Z., Gurung M., Rodrigues R.R., Padiadpu J., Newman N.K., Manes N.P. (2022). Microbiota and adipocyte mitochondrial damage in type 2 diabetes are linked by Mmp12+ macrophages. J. Exp. Med..

[23] Song M., Zhang S., Tao Z., Li J., Shi Y., Xiong Y. (2021). MMP-12 siRNA improves the homeostasis of the small intestine and metabolic dysfunction in high-fat diet feeding-induced obese mice. Biomaterials.

[24] Amor M., Bianco V., Buerger M., Lechleitner M., Vujic N., Dobrijevic A. (2023). Genetic deletion of MMP12 ameliorates cardiometabolic disease by improving insulin sensitivity, systemic inflammation, and atherosclerotic features in mice. Cardiovasc. Diabetol..

[25] Vujic N., Schlager S., Eichmann T.O., Madreiter-Sokolowski C.T., Goeritzer M., Rainer S. (2016). Monoglyceride lipase deficiency modulates endocannabinoid signaling and improves plaque stability in ApoE-knockout mice. Atherosclerosis.

[26] Korbelius M., Vujic N., Sachdev V., Obrowsky S., Rainer S., Gottschalk B. (2019). ATGL/CGI-58-Dependent hydrolysis of a lipid storage pool in murine enterocytes. Cell Rep..

[27] Kim H.S., Ryoo Z.Y., Choi S.U., Lee S. (2015). Gene expression profiles reveal effect of a high-fat diet on the development of white and brown adipose tissues. Gene.

[28] Edgar R., Domrachev M., Lash A.E. (2002). Gene expression omnibus: NCBI gene expression and hybridization array data repository. Nucleic Acids Res..

[29] Barrett T., Wilhite S.E., Ledoux P., Evangelista C., Kim I.F., Tomashevsky M. (2013). NCBI GEO: archive for functional genomics data sets--update. Nucleic Acids Res..

[30] Amor M., Diaz M., Bianco V., Svecla M., Schwarz B., Rainer S. (2024). Identification of regulatory networks and crosstalk factors in brown adipose tissue and liver of a cold-exposed cardiometabolic mouse model. Cardiovasc. Diabetol..

[31] Svecla M., Nour J., Bladergroen M.R., Nicolardi S., Zhang T., Beretta G. (2023). Impact of asialoglycoprotein receptor and mannose receptor deficiency on murine plasma N-glycome profiles. Mol. Cell Proteomics.

[32] Holman J.D., Tabb D.L., Mallick P. (2014). Employing ProteoWizard to convert raw mass spectrometry data. Curr. Protoc. Bioinformatics.

[33] Svecla M., Garrone G., Faré F., Aletti G., Norata G.D., Beretta G. (2021). DDASSQ: an open-source, multiple peptide sequencing strategy for label free quantification based on an OpenMS pipeline in the KNIME analytics platform. Proteomics.

[34] Goedhart J., Luijsterburg M.S. (2020). VolcaNoseR is a web app for creating, exploring, labeling and sharing volcano plots. Sci. Rep..

[35] Krämer A., Green J., Pollard J., Tugendreich S. (2014). Causal analysis approaches in ingenuity pathway analysis. Bioinformatics.

[36] Pagliarini D.J., Calvo S.E., Chang B., Sheth S.A., Vafai S.B., Ong S.E. (2008). A mitochondrial protein compendium elucidates complex I disease biology. Cell.

[37] Calvo S.E., Clauser K.R., Mootha V.K. (2016). MitoCarta2.0: an updated inventory of mammalian mitochondrial proteins. Nucleic Acids Res..

[38] Rath S., Sharma R., Gupta R., Ast T., Chan C., Durham T.J. (2021). MitoCarta3.0: an updated mitochondrial proteome now with sub-organelle localization and pathway annotations. Nucleic Acids Res..

[39] Rogers G.W., Brand M.D., Petrosyan S., Ashok D., Elorza A.A., Ferrick D.A. (2011). High throughput microplate respiratory measurements using minimal quantities of isolated mitochondria. PLoS One.

[40] Schindelin J., Arganda-Carreras I., Frise E., Kaynig V., Longair M., Pietzsch T. (2012). Fiji: an open-source platform for biological-image analysis. Nat. Methods.

[41] Ollion J., Cochennec J., Loll F., Escudé C., Boudier T. (2013). TANGO: a generic tool for high-throughput 3D image analysis for studying nuclear organization. Bioinformatics.

[42] Liu X., Zhu Y., Zhan S., Zhong T., Guo J., Cao J. (2022). RNA-Seq reveals miRNA role in thermogenic regulation in brown adipose tissues of goats. BMC Genomics.

[43] Lemecha M., Morino K., Imamura T., Iwasaki H., Ohashi N., Ida S. (2018). MiR-494-3p regulates mitochondrial biogenesis and thermogenesis through PGC1-α signalling in beige adipocytes. Sci. Rep..

[44] Kim E.M., Shin E.J., Choi J.H., Son H.J., Park I.S., Joh T.H. (2010). Matrix metalloproteinase-3 is increased and participates in neuronal apoptotic signaling downstream of caspase-12 during endoplasmic reticulum stress. J. Biol. Chem..

[45] Lindsey M.L., Escobar G.P., Mukherjee R., Goshorn D.K., Sheats N.J., Bruce J.A. (2006). Matrix metalloproteinase-7 affects connexin-43 levels, electrical conduction, and survival after myocardial infarction. Circulation.

[46] Zhang Z., Amorosa L.F., Coyle S.M., Macor M.A., Lubitz S.E., Carson J.L. (2015). Proteolytic cleavage of AMPKα and intracellular MMP9 expression are both required for TLR4-Mediated mTORC1 activation and HIF-1α expression in leukocytes. J. Immunol..

[47] Pulakazhi Venu V.K., Uboldi P., Dhyani A., Patrini A., Baetta R., Ferri N. (2015). Fibronectin extra domain A stabilises atherosclerotic plaques in apolipoprotein E and in LDL-receptor-deficient mice. Thromb. Haemost..

[48] Golubkov V.S., Boyd S., Savinov A.Y., Chekanov A.V., Osterman A.L., Remacle A. (2005). Membrane type-1 matrix metalloproteinase (MT1-MMP) exhibits an important intracellular cleavage function and causes chromosome instability. J. Biol. Chem..

[49] Savinov A.Y., Remacle A.G., Golubkov V.S., Krajewska M., Kennedy S., Duffy M.J. (2006). Matrix metalloproteinase 26 proteolysis of the NH2-terminal domain of the estrogen receptor beta correlates with the survival of breast cancer patients. Cancer Res..

[50] Sakamoto T., Seiki M. (2010). A membrane protease regulates energy production in macrophages by activating hypoxia-inducible factor-1 via a non-proteolytic mechanism. J. Biol. Chem..

[51] Mackert O., Wirth E.K., Sun R., Winkler J., Liu A., Renko K. (2022). Impact of metabolic stress induced by diets, aging and fasting on tissue oxygen consumption. Mol. Metab..

[52] Böhm A., Keuper M., Meile T., Zdichavsky M., Fritsche A., Häring H.U. (2020). Increased mitochondrial respiration of adipocytes from metabolically unhealthy obese compared to healthy obese individuals. Sci. Rep..

[53] Madreiter-Sokolowski C.T., Thomas C., Ristow M. (2020). Interrelation between ROS and Ca(2+) in aging and age-related diseases. Redox Biol..

[54] Zorov D.B., Juhaszova M., Sollott S.J. (2014). Mitochondrial reactive oxygen species (ROS) and ROS-induced ROS release. Physiol. Rev..

[55] Huttlin E.L., Jedrychowski M.P., Elias J.E., Goswami T., Rad R., Beausoleil S.A. (2010). A tissue-specific atlas of mouse protein phosphorylation and expression. Cell.

[56] Manetti M., Guiducci S., Romano E., Bellando-Randone S., Conforti M.L., Ibba-Manneschi L. (2012). Increased serum levels and tissue expression of matrix metalloproteinase-12 in patients with systemic sclerosis: correlation with severity of skin and pulmonary fibrosis and vascular damage. Ann. Rheum. Dis..

[57] Goncalves I., Bengtsson E., Colhoun H.M., Shore A.C., Palombo C., Natali A. (2015). Elevated plasma levels of MMP-12 are associated with atherosclerotic burden and symptomatic cardiovascular disease in subjects with type 2 diabetes. Arterioscler. Thromb. Vasc. Biol..

[58] Aquilano K., Zhou B., Brestoff J.R., Lettieri-Barbato D. (2023). Multifaceted mitochondrial quality control in brown adipose tissue. Trends Cell Biol..

[59] Henriques F., Bedard A.H., Guilherme A., Kelly M., Chi J., Zhang P. (2020). Single-Cell RNA profiling reveals adipocyte to macrophage signaling sufficient to enhance thermogenesis. Cell Rep..

[60] Rosina M., Ceci V., Turchi R., Chuan L., Borcherding N., Sciarretta F. (2022). Ejection of damaged mitochondria and their removal by macrophages ensure efficient thermogenesis in brown adipose tissue. Cell Metab..

[61] Cannon B., Nedergaard J. (2011). Nonshivering thermogenesis and its adequate measurement in metabolic studies. J. Exp. Biol..

[62] Perez-Riverol Y., Bai J., Bandla C., García-Seisdedos D., Hewapathirana S., Kamatchinathan S. (2022). The PRIDE database resources in 2022: a hub for mass spectrometry-based proteomics evidences. Nucleic Acids Res..

