# Supplementary material for: Deletion of MMP12 improves energy metabolism and brown adipose tissue function in mice prone to cardiometabolic disease
Source: J Lipid Res. 2025 Nov 26;67(1):100951. doi: 10.1016/j.jlr.2025.100951 (PMC12775818; doi:10.1016/j.jlr.2025.100951)
Supplement: Supplementary information [file mmc1.pptx]

## Slide 1
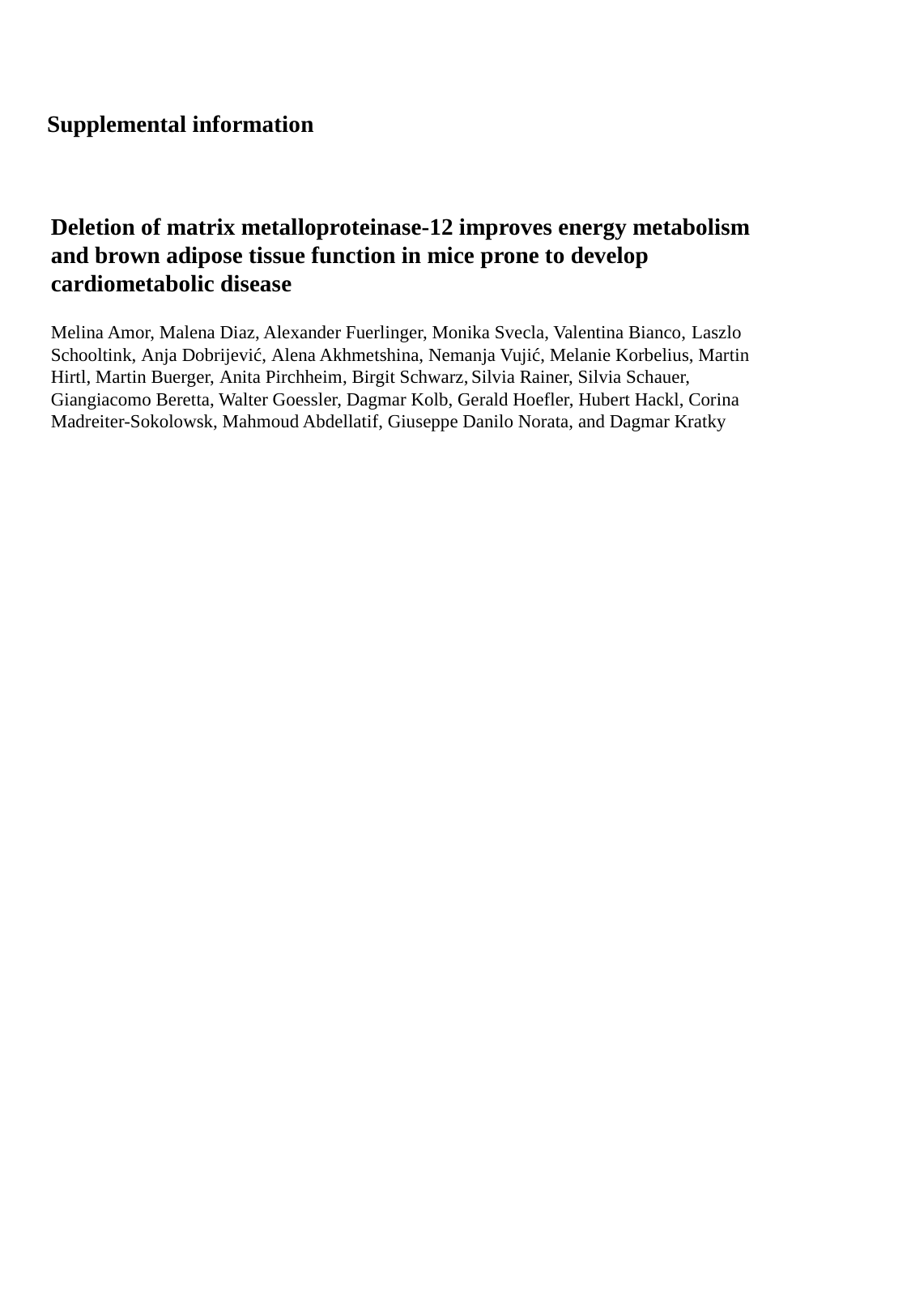

Supplemental information
Deletion of matrix metalloproteinase-12 improves energy metabolism and brown adipose tissue function in mice prone to develop cardiometabolic disease
Melina Amor, Malena Diaz, Alexander Fuerlinger, Monika Svecla, Valentina Bianco, Laszlo Schooltink, Anja Dobrijević, Alena Akhmetshina, Nemanja Vujić, Melanie Korbelius, Martin Hirtl, Martin Buerger, Anita Pirchheim, Birgit Schwarz, Silvia Rainer, Silvia Schauer, Giangiacomo Beretta, Walter Goessler, Dagmar Kolb, Gerald Hoefler, Hubert Hackl, Corina Madreiter-Sokolowsk, Mahmoud Abdellatif, Giuseppe Danilo Norata, and Dagmar Kratky

## Slide 2
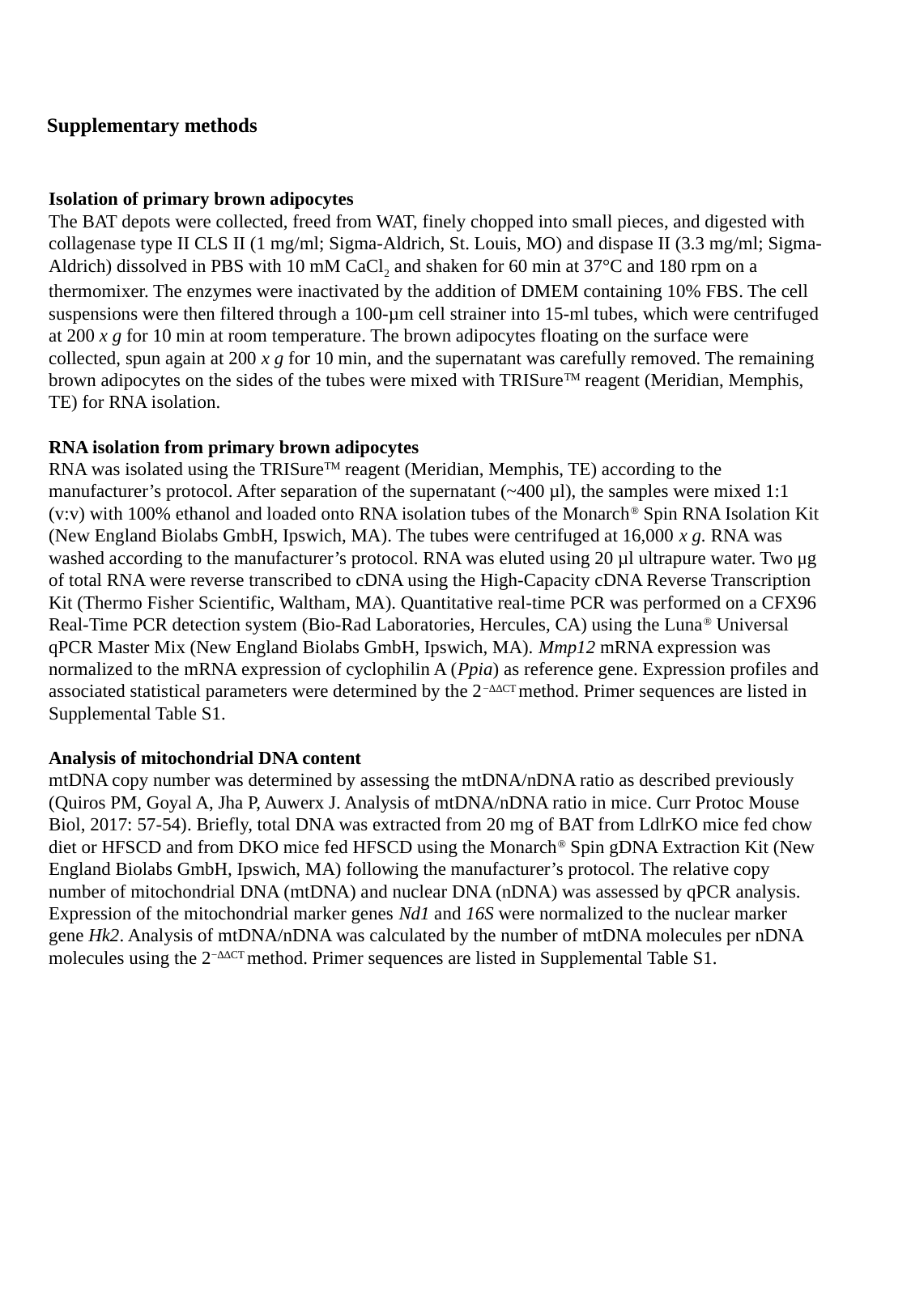

Supplementary methods
Isolation of primary brown adipocytes
The BAT depots were collected, freed from WAT, finely chopped into small pieces, and digested with collagenase type II CLS II (1 mg/ml; Sigma-Aldrich, St. Louis, MO) and dispase II (3.3 mg/ml; Sigma-Aldrich) dissolved in PBS with 10 mM CaCl2 and shaken for 60 min at 37°C and 180 rpm on a thermomixer. The enzymes were inactivated by the addition of DMEM containing 10% FBS. The cell suspensions were then filtered through a 100-µm cell strainer into 15-ml tubes, which were centrifuged at 200 x g for 10 min at room temperature. The brown adipocytes floating on the surface were collected, spun again at 200 x g for 10 min, and the supernatant was carefully removed. The remaining brown adipocytes on the sides of the tubes were mixed with TRISureTM reagent (Meridian, Memphis, TE) for RNA isolation.
RNA isolation from primary brown adipocytes
RNA was isolated using the TRISureTM reagent (Meridian, Memphis, TE) according to the manufacturer’s protocol. After separation of the supernatant (~400 µl), the samples were mixed 1:1 (v:v) with 100% ethanol and loaded onto RNA isolation tubes of the Monarch® Spin RNA Isolation Kit (New England Biolabs GmbH, Ipswich, MA). The tubes were centrifuged at 16,000 x g. RNA was washed according to the manufacturer’s protocol. RNA was eluted using 20 µl ultrapure water. Two μg of total RNA were reverse transcribed to cDNA using the High-Capacity cDNA Reverse Transcription Kit (Thermo Fisher Scientific, Waltham, MA). Quantitative real-time PCR was performed on a CFX96 Real-Time PCR detection system (Bio-Rad Laboratories, Hercules, CA) using the Luna® Universal qPCR Master Mix (New England Biolabs GmbH, Ipswich, MA). Mmp12 mRNA expression was normalized to the mRNA expression of cyclophilin A (Ppia) as reference gene. Expression profiles and associated statistical parameters were determined by the 2−ΔΔCT method. Primer sequences are listed in Supplemental Table S1.
Analysis of mitochondrial DNA content
mtDNA copy number was determined by assessing the mtDNA/nDNA ratio as described previously (Quiros PM, Goyal A, Jha P, Auwerx J. Analysis of mtDNA/nDNA ratio in mice. Curr Protoc Mouse Biol, 2017: 57-54). Briefly, total DNA was extracted from 20 mg of BAT from LdlrKO mice fed chow diet or HFSCD and from DKO mice fed HFSCD using the Monarch® Spin gDNA Extraction Kit (New England Biolabs GmbH, Ipswich, MA) following the manufacturer’s protocol. The relative copy number of mitochondrial DNA (mtDNA) and nuclear DNA (nDNA) was assessed by qPCR analysis. Expression of the mitochondrial marker genes Nd1 and 16S were normalized to the nuclear marker gene Hk2. Analysis of mtDNA/nDNA was calculated by the number of mtDNA molecules per nDNA molecules using the 2−ΔΔCT method. Primer sequences are listed in Supplemental Table S1.

## Slide 3
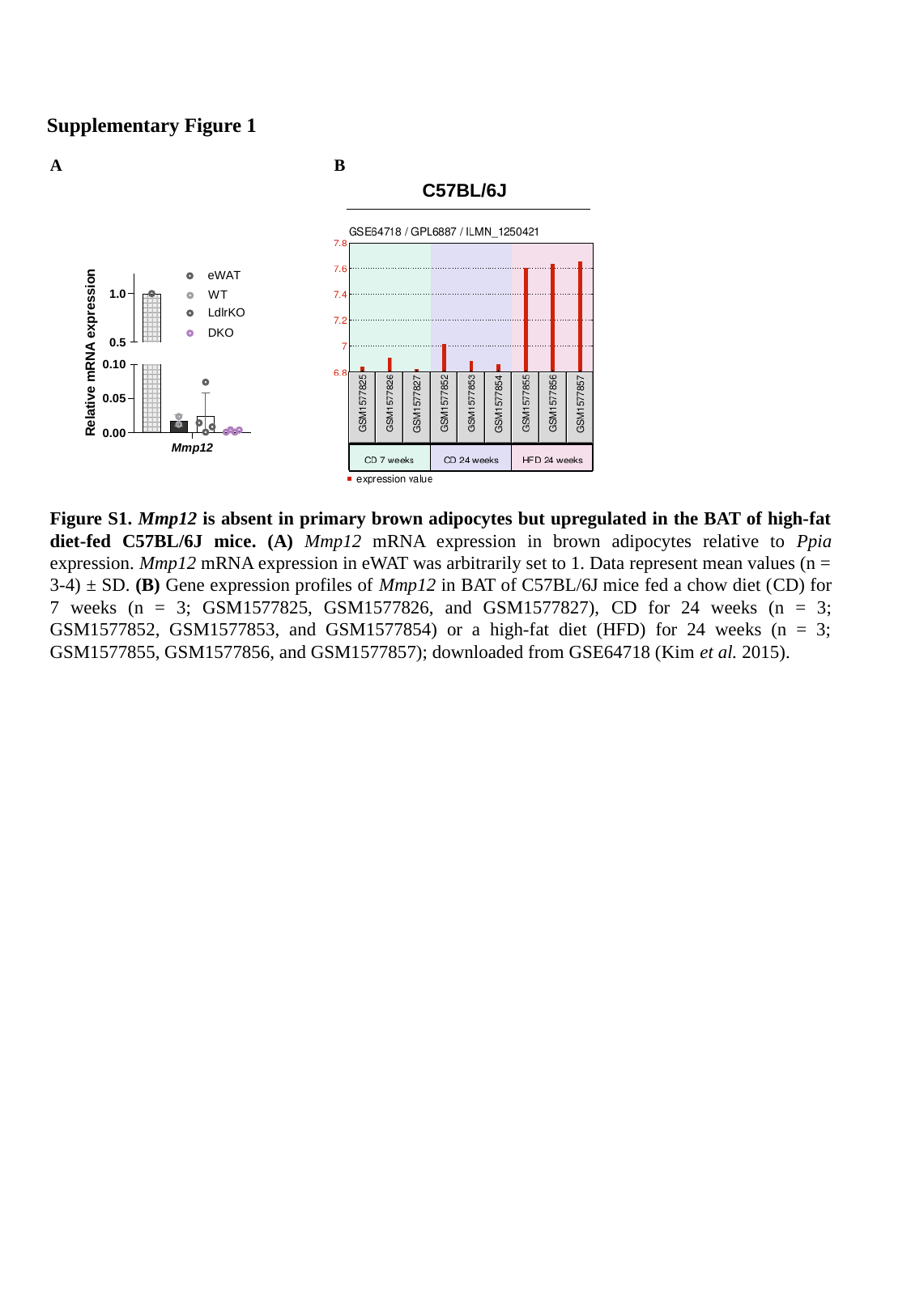

Supplementary Figure 1
A
B
C57BL/6J
Figure S1. Mmp12 is absent in primary brown adipocytes but upregulated in the BAT of high-fat diet-fed C57BL/6J mice. (A) Mmp12 mRNA expression in brown adipocytes relative to Ppia expression. Mmp12 mRNA expression in eWAT was arbitrarily set to 1. Data represent mean values (n = 3-4) ± SD. (B) Gene expression profiles of Mmp12 in BAT of C57BL/6J mice fed a chow diet (CD) for 7 weeks (n = 3; GSM1577825, GSM1577826, and GSM1577827), CD for 24 weeks (n = 3; GSM1577852, GSM1577853, and GSM1577854) or a high-fat diet (HFD) for 24 weeks (n = 3; GSM1577855, GSM1577856, and GSM1577857); downloaded from GSE64718 (Kim et al. 2015).

## Slide 4
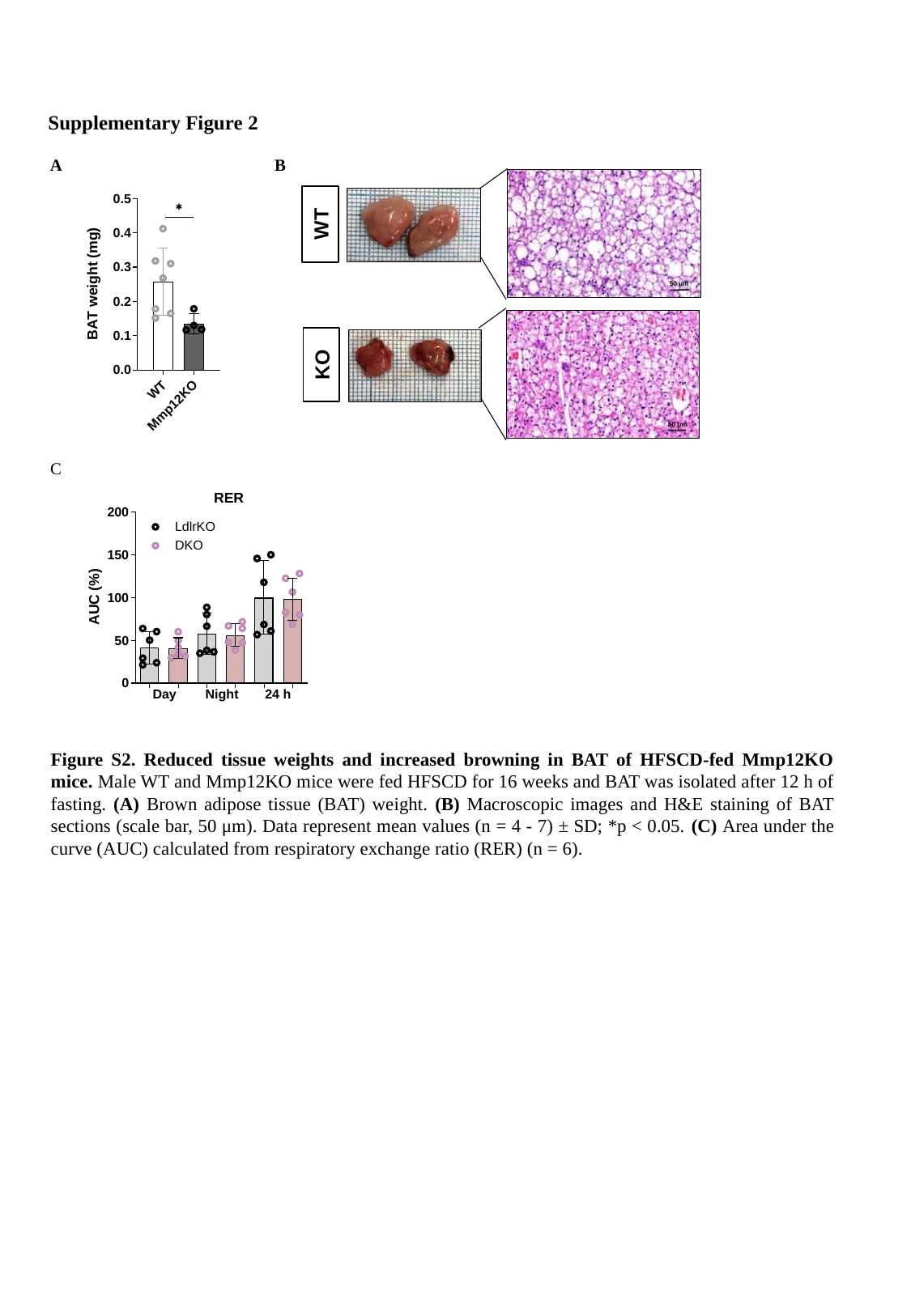

Supplementary Figure 2
A
B
WT
KO
50 μm
50 μm
C
Figure S2. Reduced tissue weights and increased browning in BAT of HFSCD-fed Mmp12KO mice. Male WT and Mmp12KO mice were fed HFSCD for 16 weeks and BAT was isolated after 12 h of fasting. (A) Brown adipose tissue (BAT) weight. (B) Macroscopic images and H&E staining of BAT sections (scale bar, 50 μm). Data represent mean values (n = 4 - 7) ± SD; *p < 0.05. (C) Area under the curve (AUC) calculated from respiratory exchange ratio (RER) (n = 6).

## Slide 5
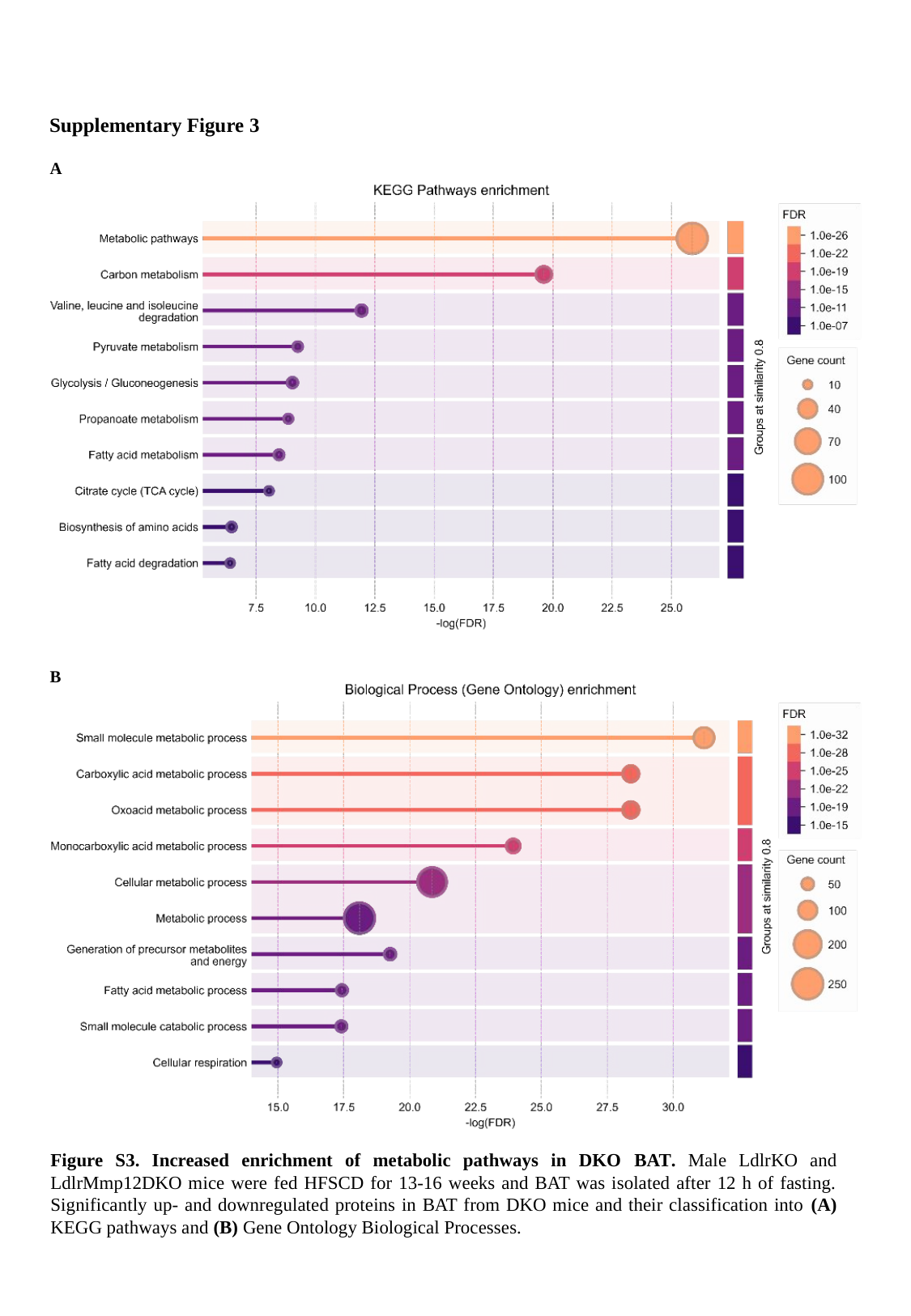

Supplementary Figure 3
A
B
Figure S3. Increased enrichment of metabolic pathways in DKO BAT. Male LdlrKO and LdlrMmp12DKO mice were fed HFSCD for 13-16 weeks and BAT was isolated after 12 h of fasting. Significantly up- and downregulated proteins in BAT from DKO mice and their classification into (A) KEGG pathways and (B) Gene Ontology Biological Processes.

## Slide 6
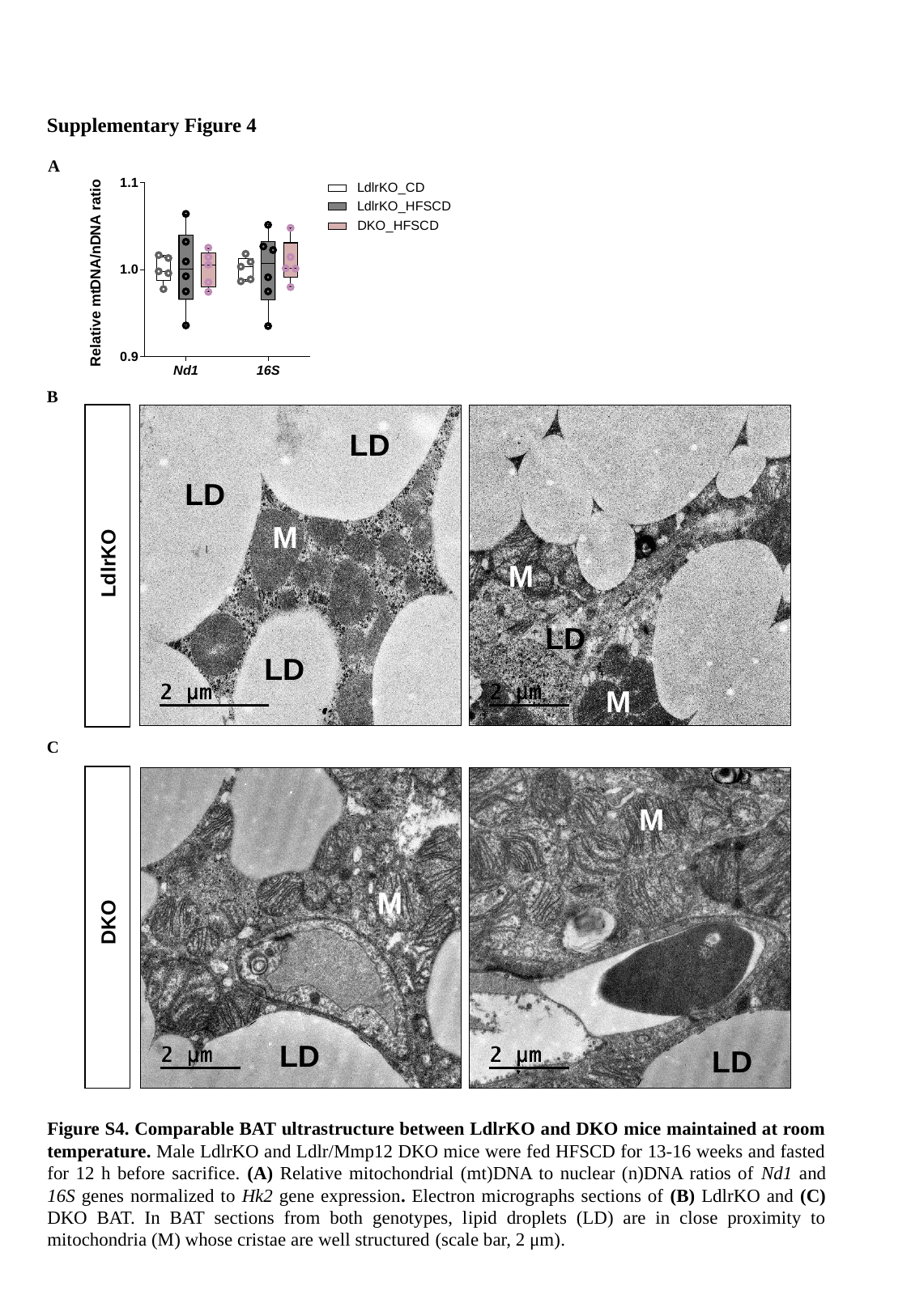

Supplementary Figure 4
A
B
LD
LD
M
LdlrKO
M
LD
LD
M
C
M
M
M
DKO
LD
LD
Figure S4. Comparable BAT ultrastructure between LdlrKO and DKO mice maintained at room temperature. Male LdlrKO and Ldlr/Mmp12 DKO mice were fed HFSCD for 13-16 weeks and fasted for 12 h before sacrifice. (A) Relative mitochondrial (mt)DNA to nuclear (n)DNA ratios of Nd1 and 16S genes normalized to Hk2 gene expression. Electron micrographs sections of (B) LdlrKO and (C) DKO BAT. In BAT sections from both genotypes, lipid droplets (LD) are in close proximity to mitochondria (M) whose cristae are well structured (scale bar, 2 μm).

## Slide 7
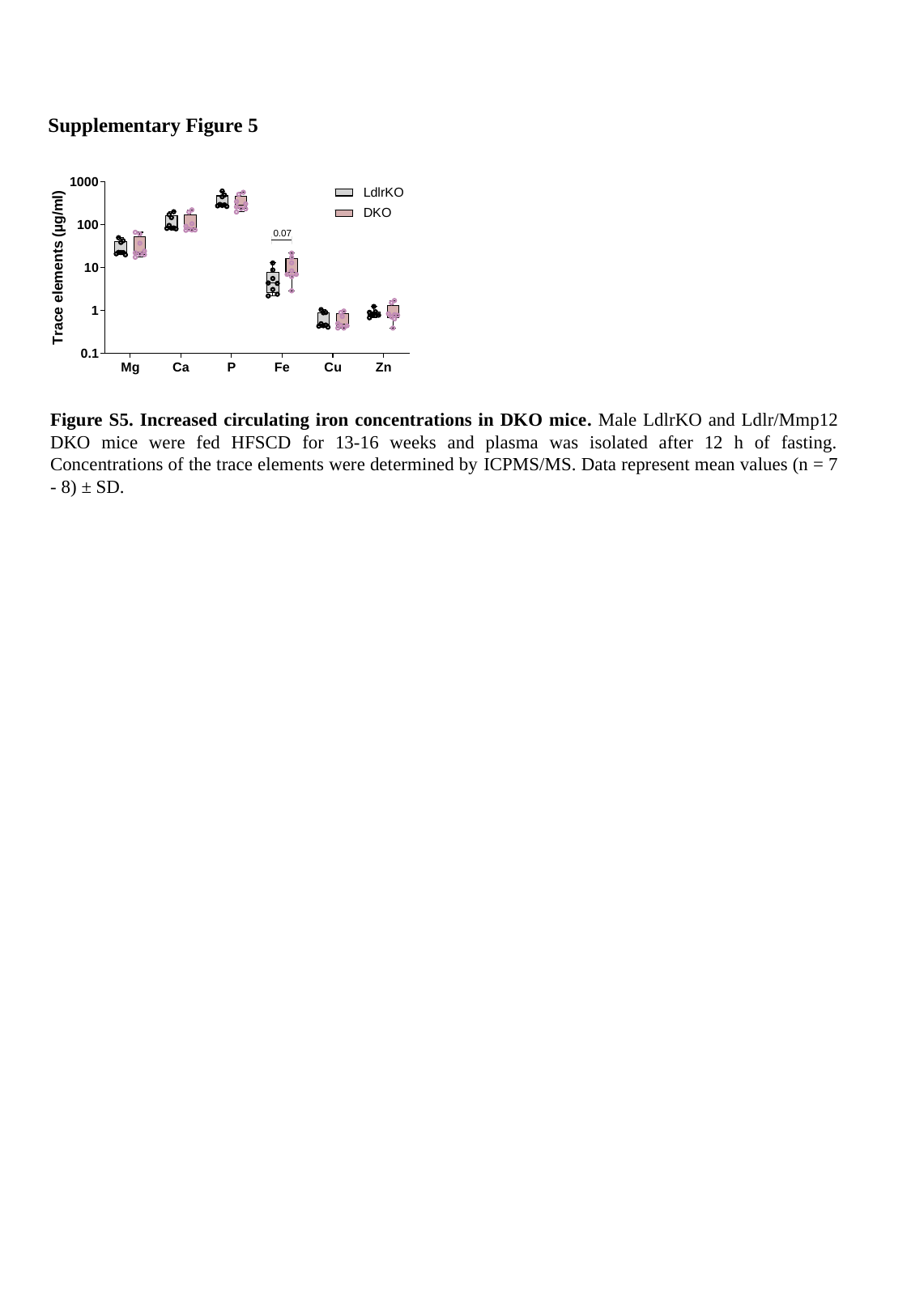

Supplementary Figure 5
Figure S5. Increased circulating iron concentrations in DKO mice. Male LdlrKO and Ldlr/Mmp12 DKO mice were fed HFSCD for 13-16 weeks and plasma was isolated after 12 h of fasting. Concentrations of the trace elements were determined by ICPMS/MS. Data represent mean values (n = 7 - 8) ± SD.

## Slide 8
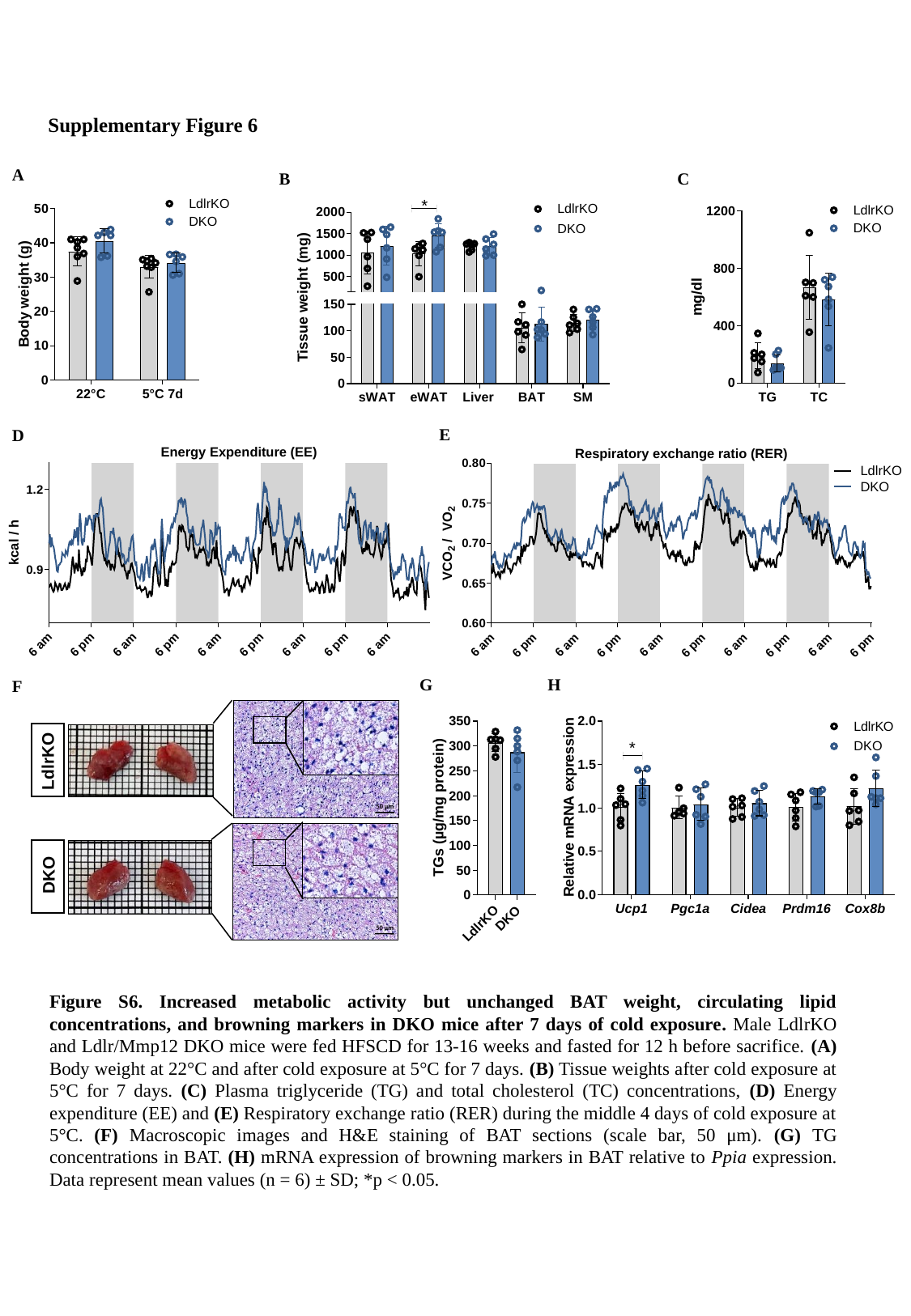

Supplementary Figure 6
A
C
B
E
D
H
G
F
LdlrKO
50 µm
DKO
50 µm
Figure S6. Increased metabolic activity but unchanged BAT weight, circulating lipid concentrations, and browning markers in DKO mice after 7 days of cold exposure. Male LdlrKO and Ldlr/Mmp12 DKO mice were fed HFSCD for 13-16 weeks and fasted for 12 h before sacrifice. (A) Body weight at 22°C and after cold exposure at 5°C for 7 days. (B) Tissue weights after cold exposure at 5°C for 7 days. (C) Plasma triglyceride (TG) and total cholesterol (TC) concentrations, (D) Energy expenditure (EE) and (E) Respiratory exchange ratio (RER) during the middle 4 days of cold exposure at 5°C. (F) Macroscopic images and H&E staining of BAT sections (scale bar, 50 μm). (G) TG concentrations in BAT. (H) mRNA expression of browning markers in BAT relative to Ppia expression. Data represent mean values (n = 6) ± SD; *p < 0.05.

## Slide 9
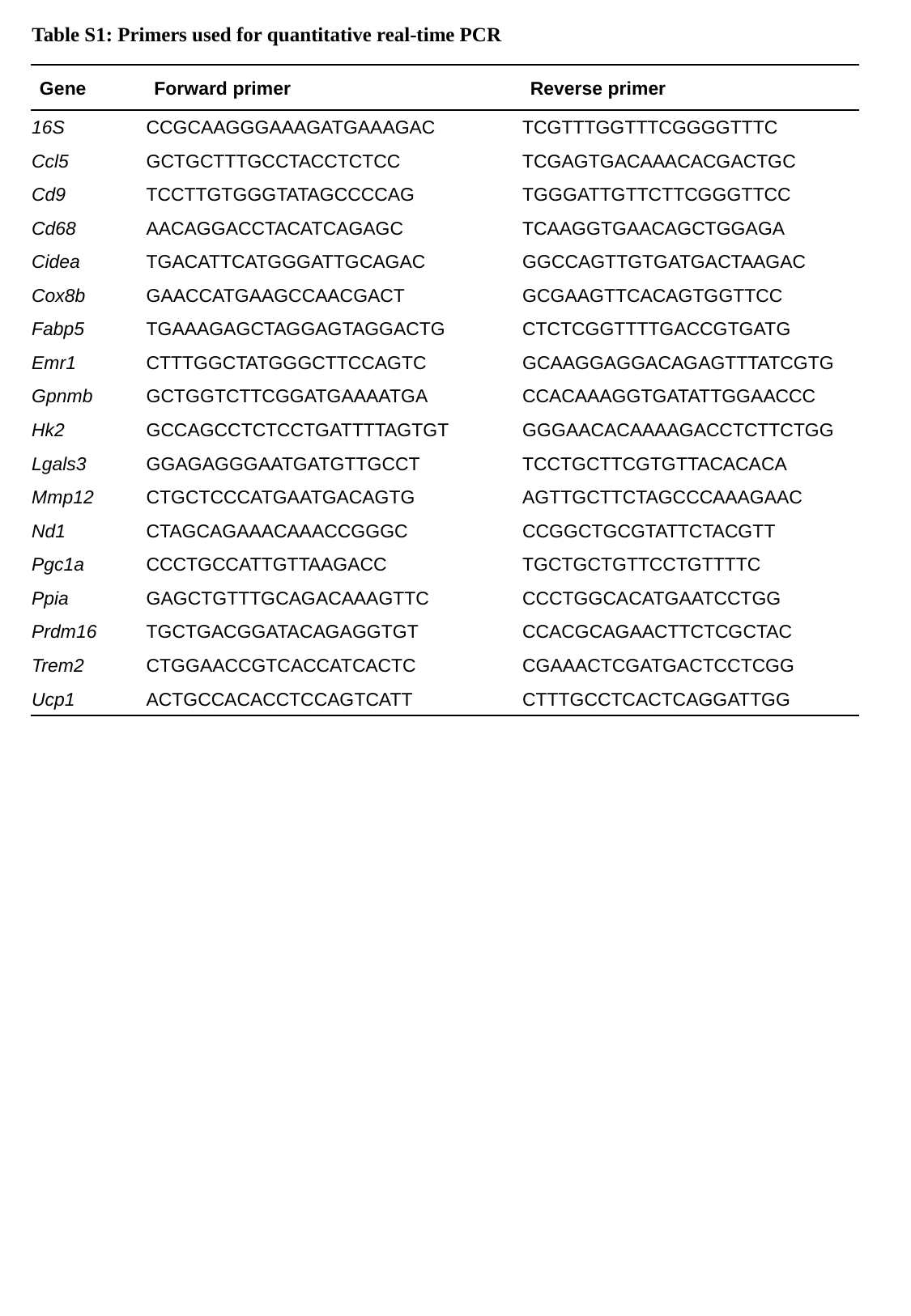

Table S1: Primers used for quantitative real-time PCR
| Gene | Forward primer | Reverse primer |
| --- | --- | --- |
| 16S | CCGCAAGGGAAAGATGAAAGAC | TCGTTTGGTTTCGGGGTTTC |
| Ccl5 | GCTGCTTTGCCTACCTCTCC | TCGAGTGACAAACACGACTGC |
| Cd9 | TCCTTGTGGGTATAGCCCCAG | TGGGATTGTTCTTCGGGTTCC |
| Cd68 | AACAGGACCTACATCAGAGC | TCAAGGTGAACAGCTGGAGA |
| Cidea | TGACATTCATGGGATTGCAGAC | GGCCAGTTGTGATGACTAAGAC |
| Cox8b | GAACCATGAAGCCAACGACT | GCGAAGTTCACAGTGGTTCC |
| Fabp5 | TGAAAGAGCTAGGAGTAGGACTG | CTCTCGGTTTTGACCGTGATG |
| Emr1 | CTTTGGCTATGGGCTTCCAGTC | GCAAGGAGGACAGAGTTTATCGTG |
| Gpnmb | GCTGGTCTTCGGATGAAAATGA | CCACAAAGGTGATATTGGAACCC |
| Hk2 | GCCAGCCTCTCCTGATTTTAGTGT | GGGAACACAAAAGACCTCTTCTGG |
| Lgals3 | GGAGAGGGAATGATGTTGCCT | TCCTGCTTCGTGTTACACACA |
| Mmp12 | CTGCTCCCATGAATGACAGTG | AGTTGCTTCTAGCCCAAAGAAC |
| Nd1 | CTAGCAGAAACAAACCGGGC | CCGGCTGCGTATTCTACGTT |
| Pgc1a | CCCTGCCATTGTTAAGACC | TGCTGCTGTTCCTGTTTTC |
| Ppia | GAGCTGTTTGCAGACAAAGTTC | CCCTGGCACATGAATCCTGG |
| Prdm16 | TGCTGACGGATACAGAGGTGT | CCACGCAGAACTTCTCGCTAC |
| Trem2 | CTGGAACCGTCACCATCACTC | CGAAACTCGATGACTCCTCGG |
| Ucp1 | ACTGCCACACCTCCAGTCATT | CTTTGCCTCACTCAGGATTGG |
